# Supplementary material for: Identification of de novo variants from parent-proband duos via long-read sequencing
Source: Am J Hum Genet. 2026 Mar 5;113(3):437–52. doi: 10.1016/j.ajhg.2026.02.006 (PMC12987547; doi:10.1016/j.ajhg.2026.02.006)
Supplement: Document S1. Figures S1–S19 and Table S2 [file mmc1.pdf]

The American Journal of Human Genetics, Volume *113*

Supplemental information

Identification of *de novo* variants from parent-proband duos via long-read sequencing

Leandros Boukas, Emmanuèle C. Délot, Georgia Pitsava, Christine Lambert, Cairbre Fanslow, Primo Baybayan, Sami Belhadj, Bojan Losic, John Harting, Krista Bluske, Jonathan LoTempio, Huda B. Al-Kouatly, Rachid Karam, William J. Rowell, Changrui Xiao, Eric Vilain, and Seth I. Berger

Supplemental Figures

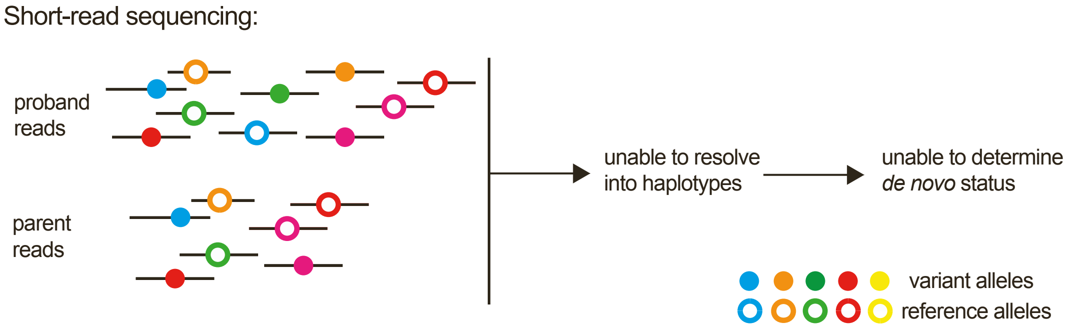

Figure S1. Cartoon representation of short reads. Like Figure 1A, but depicting short reads instead of long reads. Because of their short length, they (typically) do not contain more than one variant allele, thus precluding read-backed phasing and haplotype reconstruction.

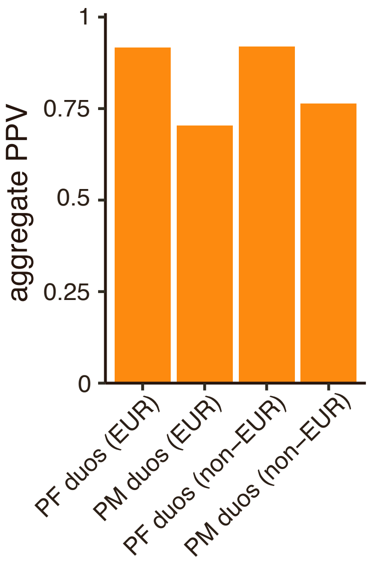

Figure S2. No major difference in the positive predictive value of *duoNovo* in probands of European vs non-European ancestry. Aggregate (across all duos) positive predictive value, shown separately for duos with probands of European versus non-European ancestry. Ancestry was calculated using Somalier[38]. Non-European ancestry groups include African (2 probands), Admixed American (17 probands), East Asian (3 probands), and South Asian (5 probands).

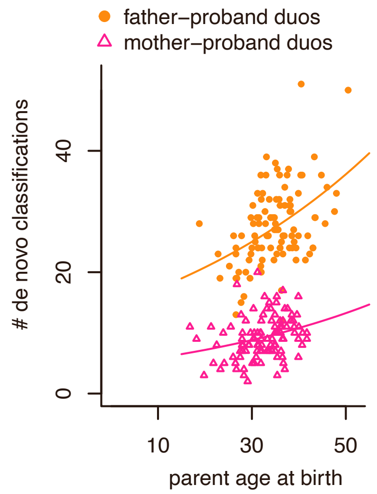

Figure S3. Paternal age positively correlates with the number of *de novo* classifications from father-proband duos. Each point corresponds to a duo. Trend lines were plotted after fitting Poisson regression models using the log link. As seen in previous studies[8], there is also a significant, but weaker, effect of maternal age.

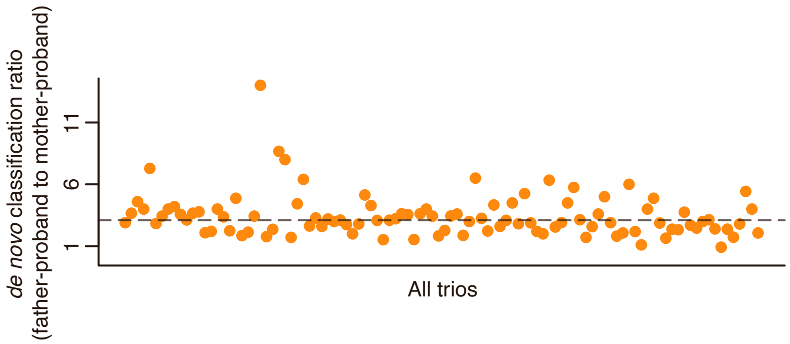

Figure S4. *duoNovo* detects more *de novo* variants from father-proband duos compared to mother-proband duos. Each point corresponds to a trio and its position on the y axis corresponds to the ratio of the number of *de novo* classifications from the father-proband duo to the number of *de novo* classifications from the mother-proband duo. The dashed horizontal line corresponds to the median across all trios.

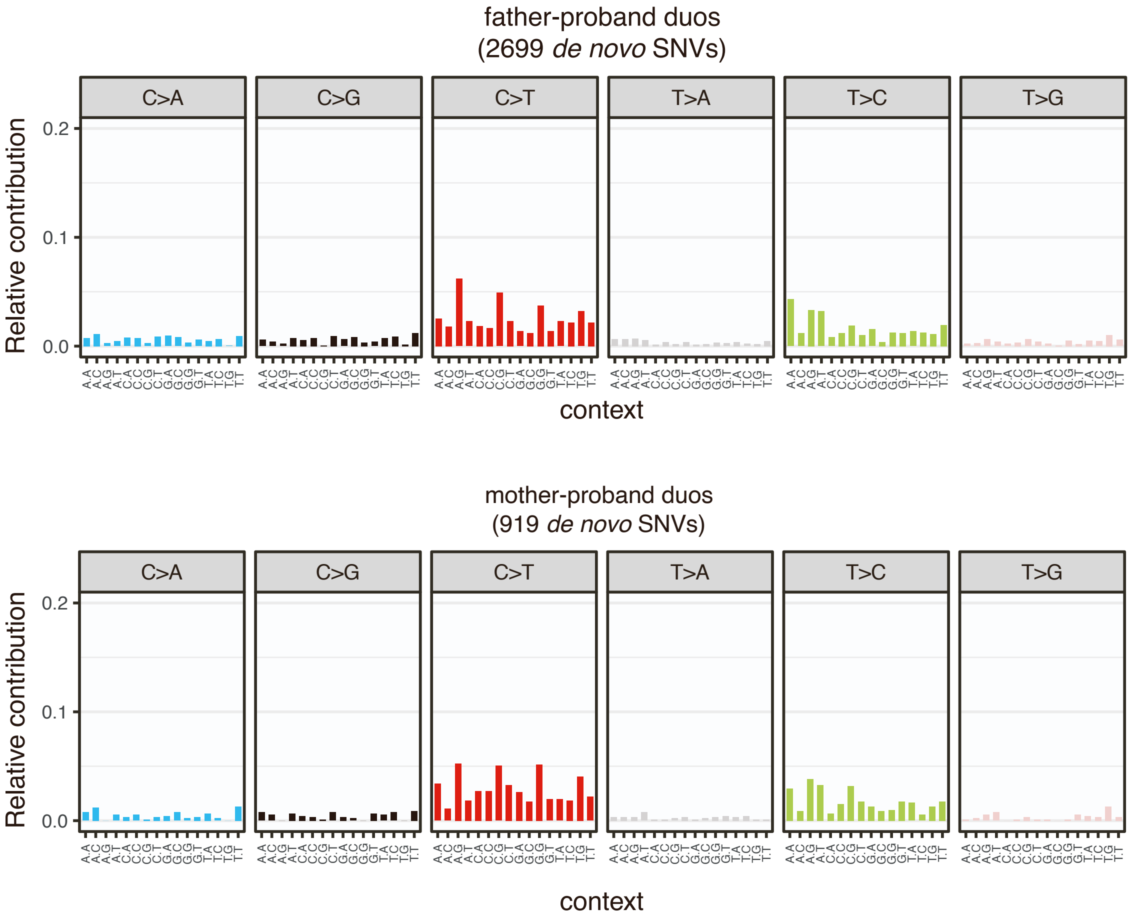

Figure S5. The *de novo* single nucleotide variants identified by *duoNovo* fall into expected mutation subtypes. The relative contributions were calculated after pooling all single nucleotide variants classified as *de novo* from the father-proband duos (top) and the mother-proband duos (bottom).

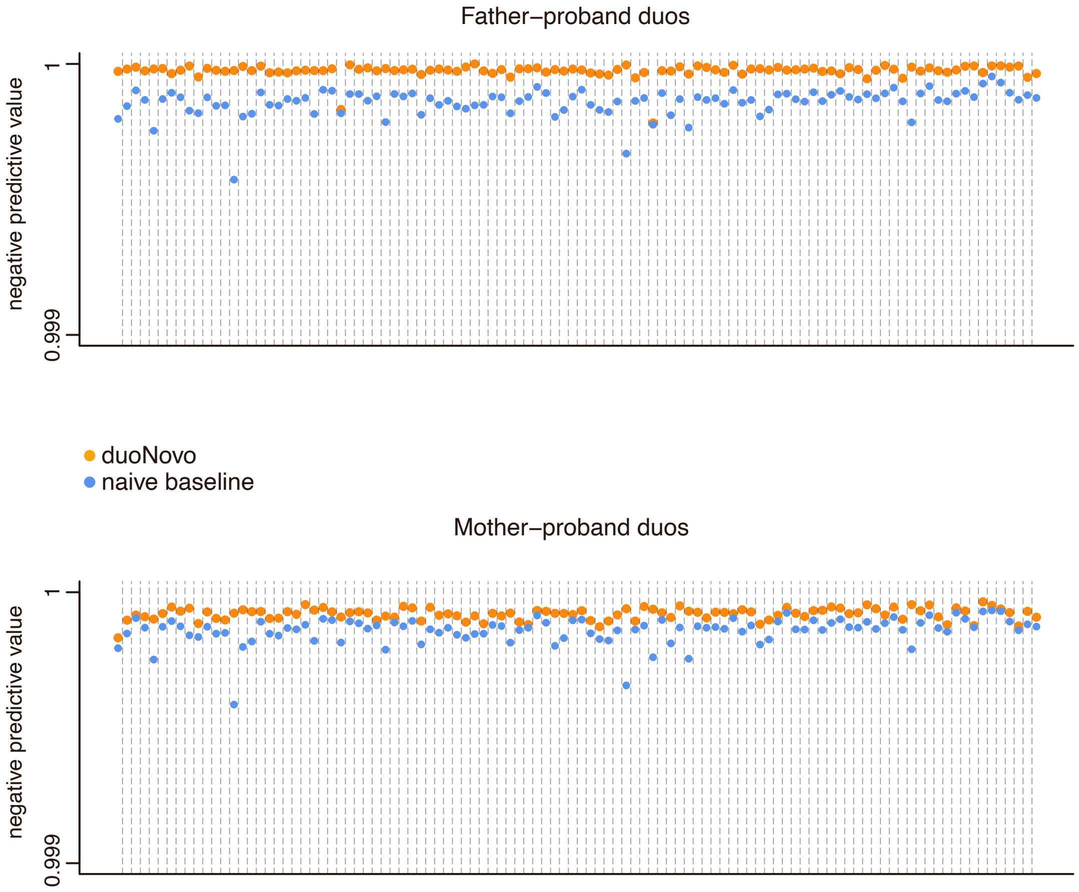

Figure S6. *duoNovo* has higher negative predictive value than the naive baseline (classifying every variant as non-*de novo*). The negative predictive value (y axis) of *duoNovo* (orange points) and of the naive baseline approach (blue points) for each father-proband and mother-proband duo.

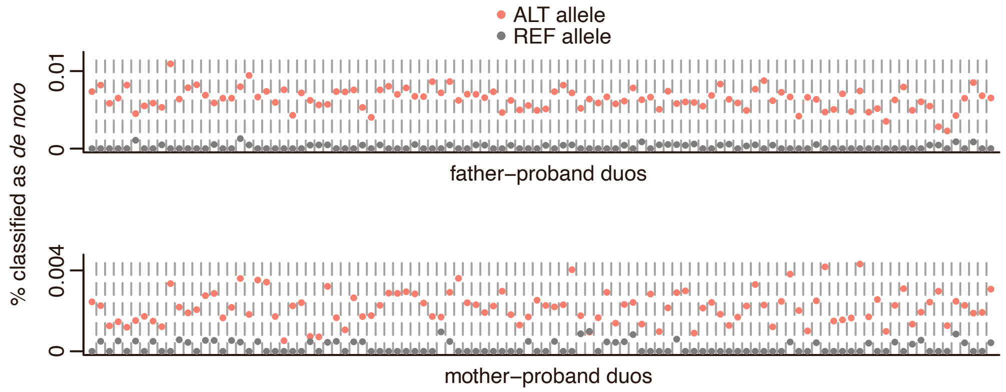

Figure S7. ***duoNovo*** **very rarely classifies candidate reference alleles as** ***de novo*****.** Candidate alternative (ALT) alleles were identified by finding positions where the proband had the "1|0" or "0|1" genotype and the parent had the "0/0" genotype. Conversely, candidate reference (REF) alleles were identified by finding positions where the proband had the "1|0" or "0|1" genotype and the parent had the "1/1" genotype. Positions were either the proband or the parent failed QC filters (Methods) were excluded.

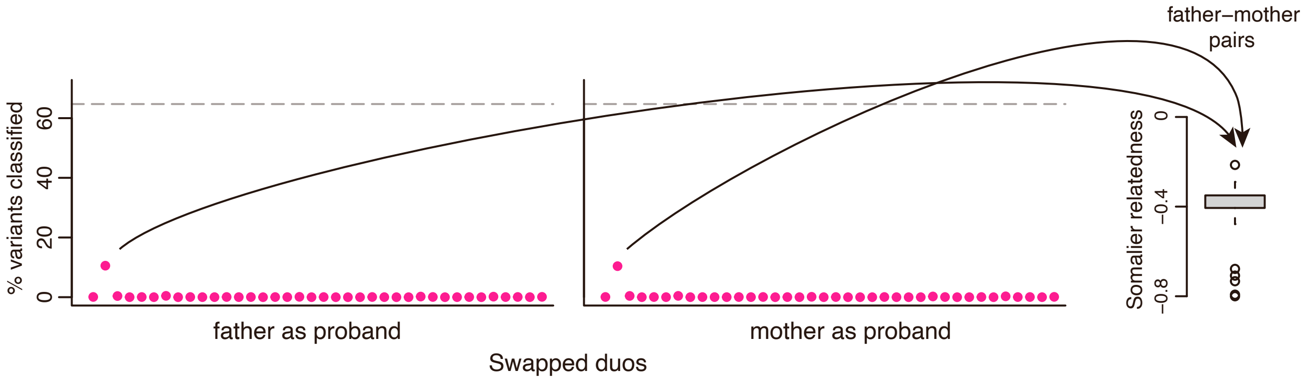

Figure S8. ***duoNovo*** **does not classify variants from swapped duos.** Each point corresponds to a swapped duo, and its position on the y axis indicates the percentage of variants that received a classification (either *de novo* or on the non-sequenced parent haplotype). Each swapped duo consists of the two parents instead of a parent and the proband; *duoNovo* was applied to each swapped duo by either treating the father as the proband (left) or the mother as the proband (right). The dashed horizontal line corresponds to the median percentage of variants that received a classification across all regular (father-proband or mother-proband) duos. The rightmost panel depicts the distribution of relatedness coefficients between all the fathers and mothers (each point corresponds to a father-mother pair), showing that the outlier swapped duo is the one with the highest relatedness coefficient.

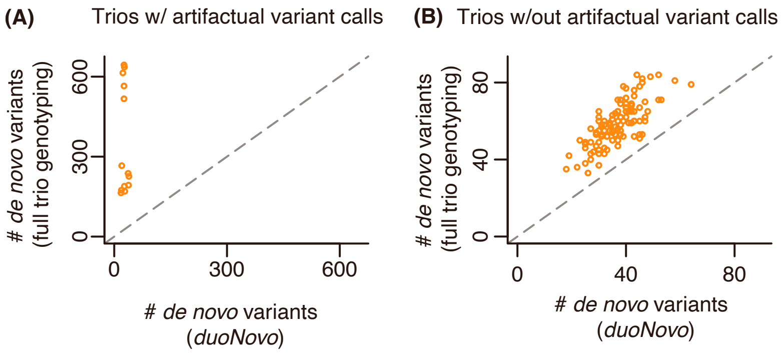

Figure S9. *duoNovo* does not classify artifactual candidate variants as *de novo*. (a) Each point corresponds to a trio with multiple artifactual variant calls generated during joint variant calling (Methods). The x-axis depicts the number of *duoNovo*'s *de novo* classifications after summing classifications from father-proband and mother-proband duos. The y-axis depicts the number of *de novo* variant calls using the standard genotype-driven approach. The dashed line is the $y = x$ line. **(b)** Like **(a)**, but for trios without artifactual variant calls. Unlike **(a)**, there is a strong correlation between the number of *de novo* variant calls using the standard genotype-driven approach and the number of total (summing father-proband and mother-proband duos) *de nono* classifications from *duoNovo*.

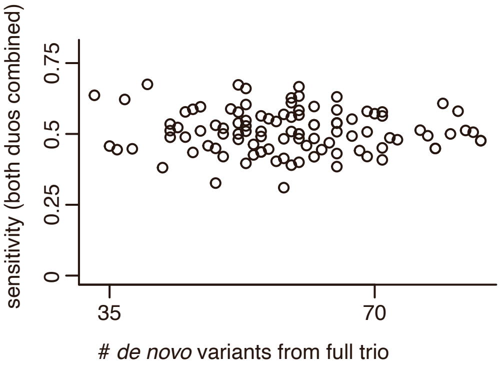

Figure S10. The relationship between *duoNovo*'s sensitivity (when combining *de novo* variants detected from father-proband and mother-proband duos) and the number of ground truth *de novo* variants detected using the standard genotype-driven approach from the full trio. Each point corresponds to a trio. The calculation of sensitivity includes trio *de novo* variants that did not receive a classification by *duoNovo* because they failed QC filtesr.

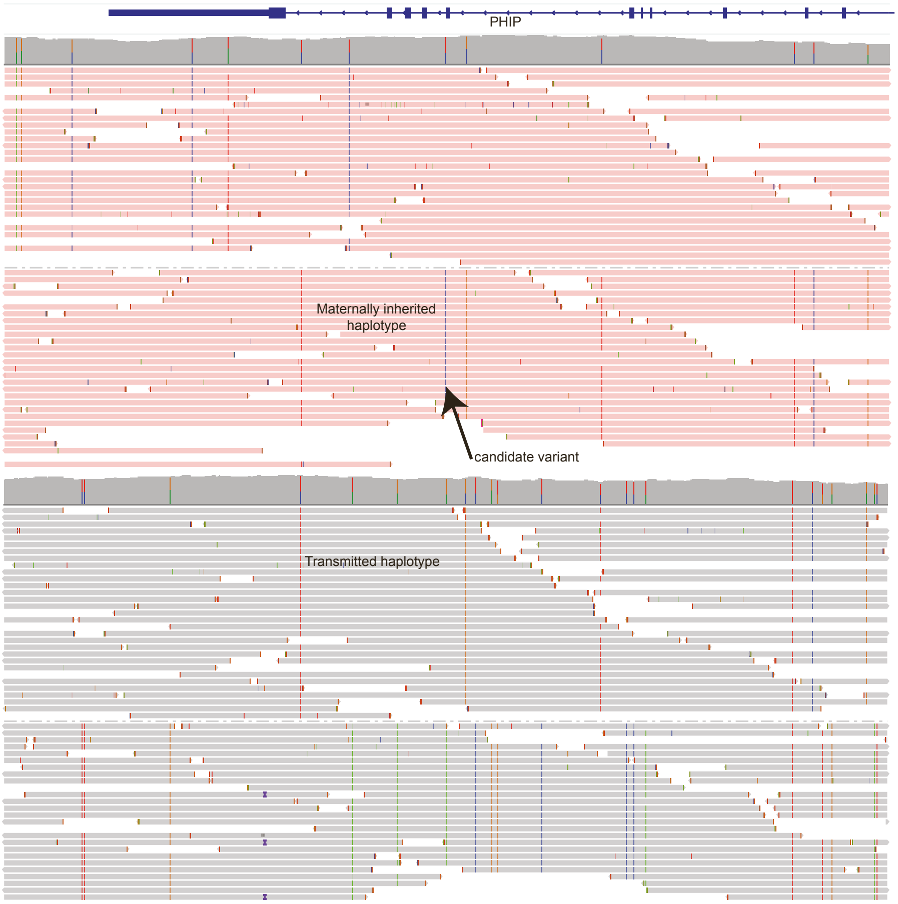

Figure S11. IGV snapshot of the sequencing reads from the proband harboring the *PHIP* intronic variant classified as *de novo* and the mother. Proband reads are shown in pink at the top and are grouped according to phase; the dashed line in the middle separates the two groups. Similarly, the maternal sequencing reads are shown in gray at the bottom grouped according to phase, with the dashed gray line separating the two groups.

Proband haplotypes

0 -- 1 -- 0 -- 1 -- 0 -- 0 -- 0 -- 1 -- 0 -- 0
0 -- 0 -- 1 -- 0 -- 1 -- 1 -- 1 -- 0 -- 1 -- 0

Parent haplotypes

0 -- 1 -- 0 -- 1 -- 0 -- 0 -- 0 -- 1 -- 0 -- 0
1 -- 0 -- 1 -- 1 -- 0 -- 0 -- 0 -- 0 -- 1 -- 1

Figure S12. Different types of genotypes that determine the Hamming distance between a proband and a parent haplotype. Red – positions heterozygous in the proband and homozygous in the parent. Blue – positions heterozygous in both proband and parent. Cyan – positions homozygous in the proband and heterozygous in the parent. The haplotypes are depicted as binary strings, with 1 indicating the variant allele and 0 the reference allele at each position.

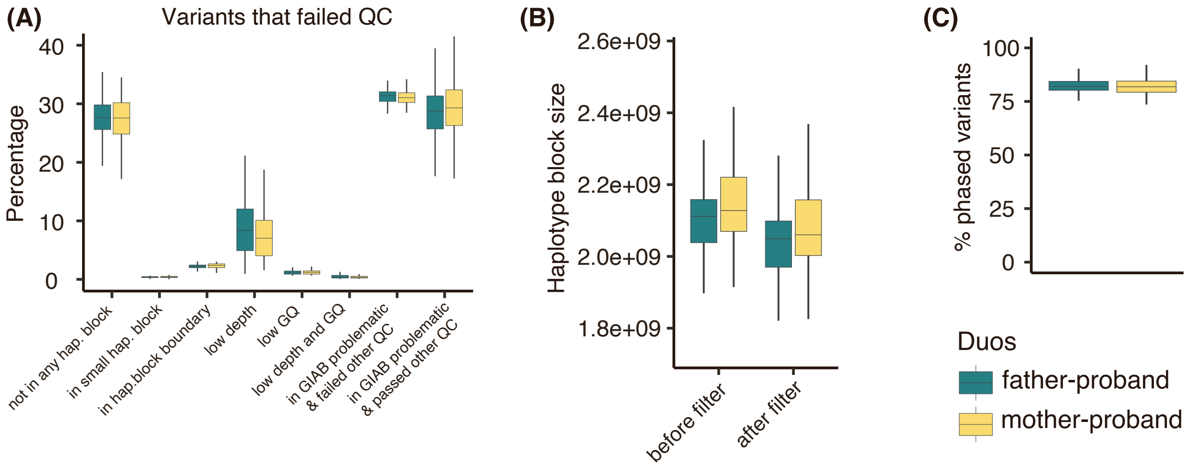

Figure S13. Lack of phasing and presence within a GIAB problematic region are the main reasons variants do not pass *duoNovo*'s QC steps. (a) Distributions (across father-proband and mother-proband duos) of the fraction of candidate variants that failed different QC steps. For each duo, each fraction is calculated among all variants that failed QC. Haplotype blocks refer to harmonized parent-proband phasing sets (Methods). **(b)** The distribution of the cumulative size of all haplotype blocks before and after applying the two relevant filters (removing haplotype blocks with size $\leq$ 10 kb and trimming the boundaries, i.e. 2 kb on either side). **(c)** The distribution of the total fraction of candidate variants that were phased across father-proband and mother-proband duos, irrespectively of other filters such as sequencing depth and GQ.

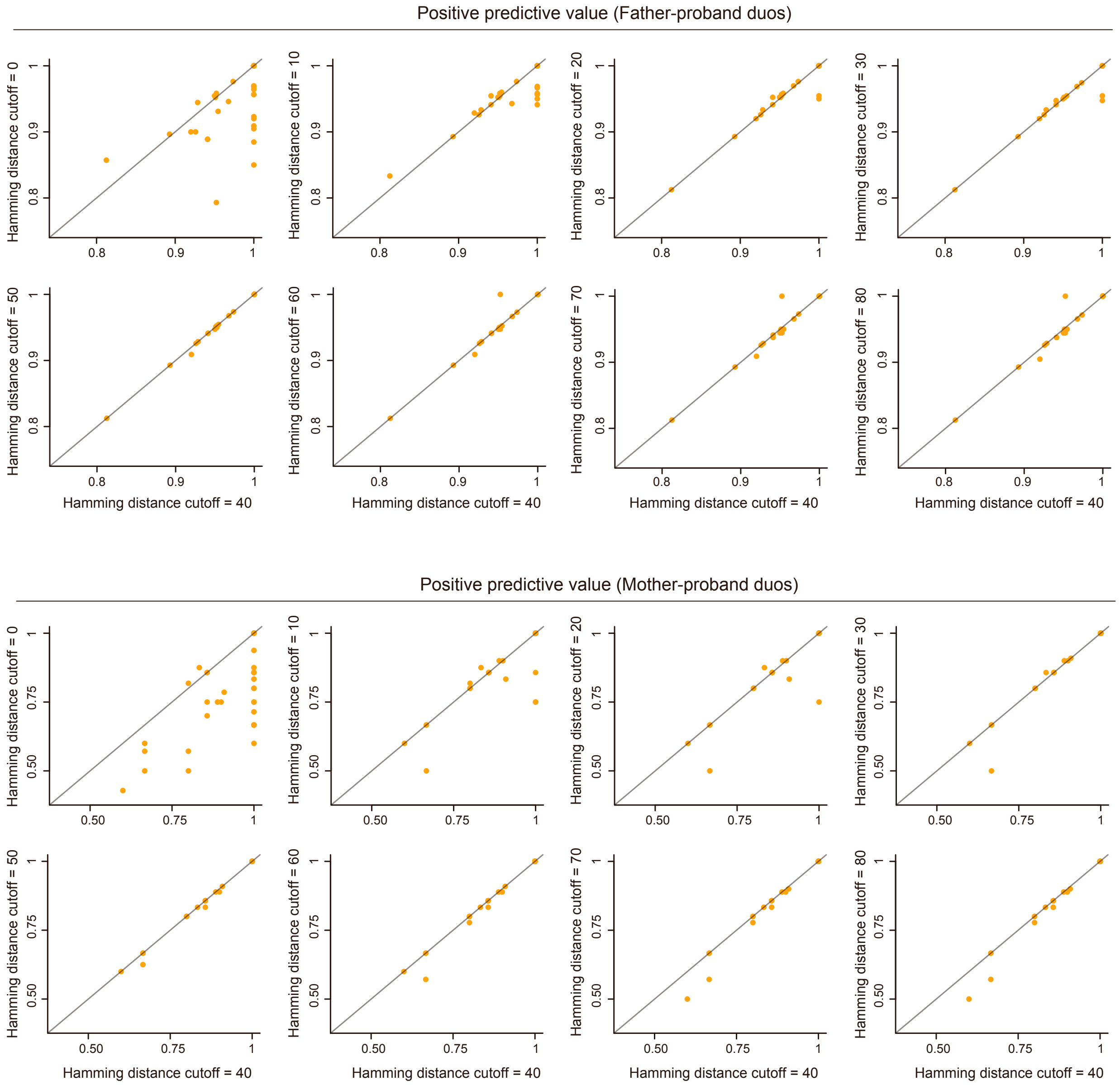

Figure S14. The impact of the Hamming distance threshold for determining dissimilarity between a pair of haplotype blocks on the positive predictive value. Each point corresponds to a duo. The x-axis shows the positive predictive value (PPV) when using a Hamming distance threshold of 40 (default), and the y axis shows the PPV when using different Hamming distance thresholds. There is some degree of overplotting, due to duos that have the same or highly similar PPV.

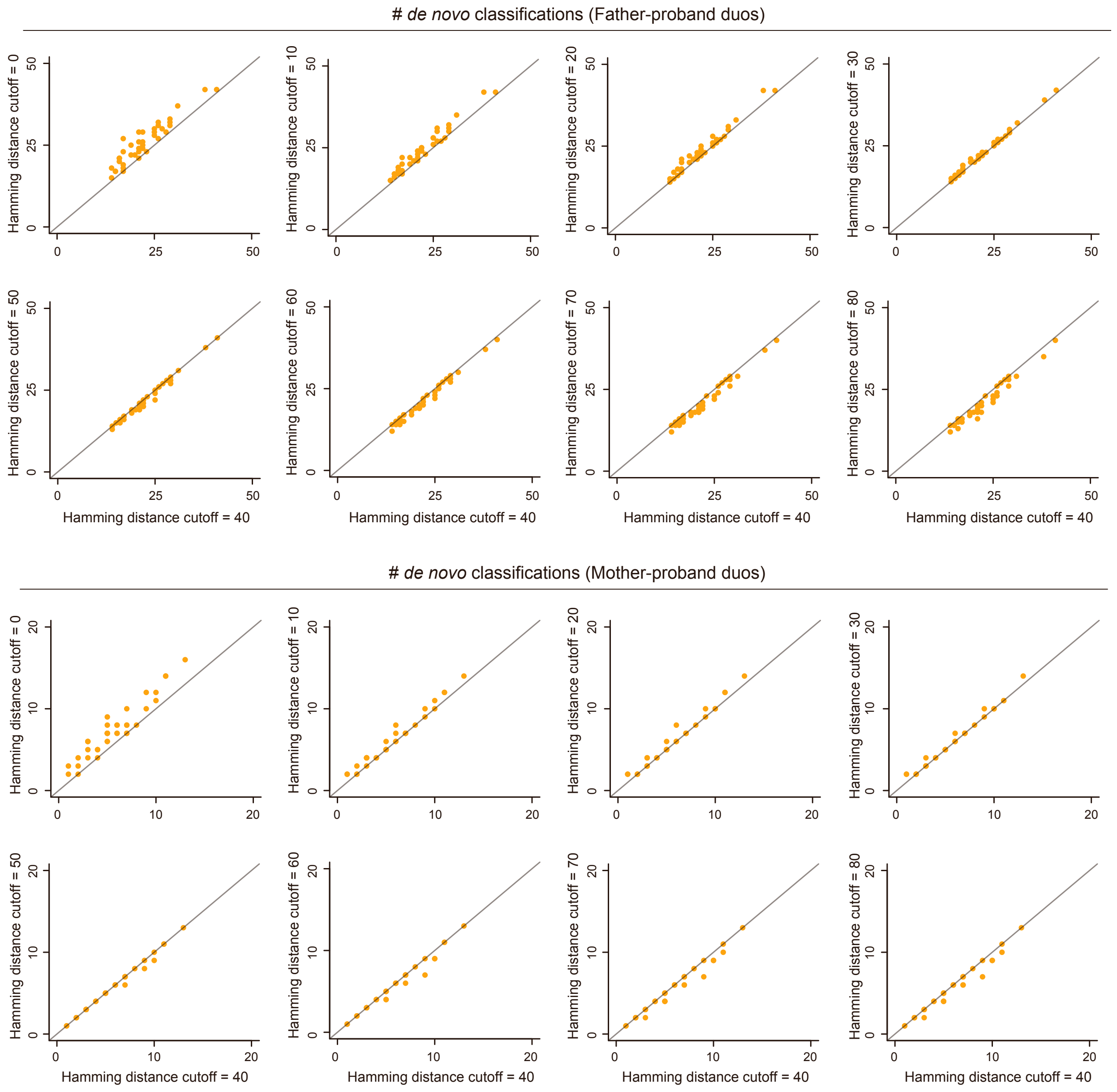

Figure S15. The impact of the Hamming distance threshold for determining dissimilarity between a pair of haplotype blocks on the number of *de novo* classifications. Like Figure S14, but depicting the number of *de novo* classifications instead of the PPV.

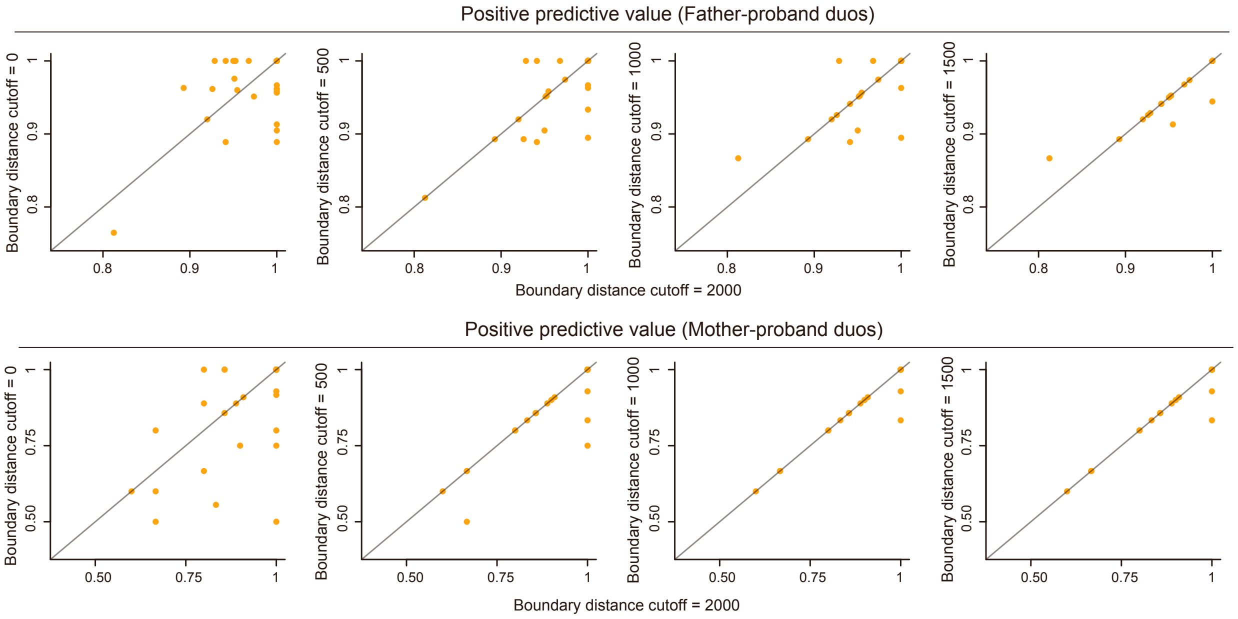

Figure S16. The impact of the distance threshold from haplotype block boundaries on the positive predictive value. Like Figure S14, but showing the impact of the threshold for the distance from the haplotype block boundaries (start/end coordinates) on the PPV.

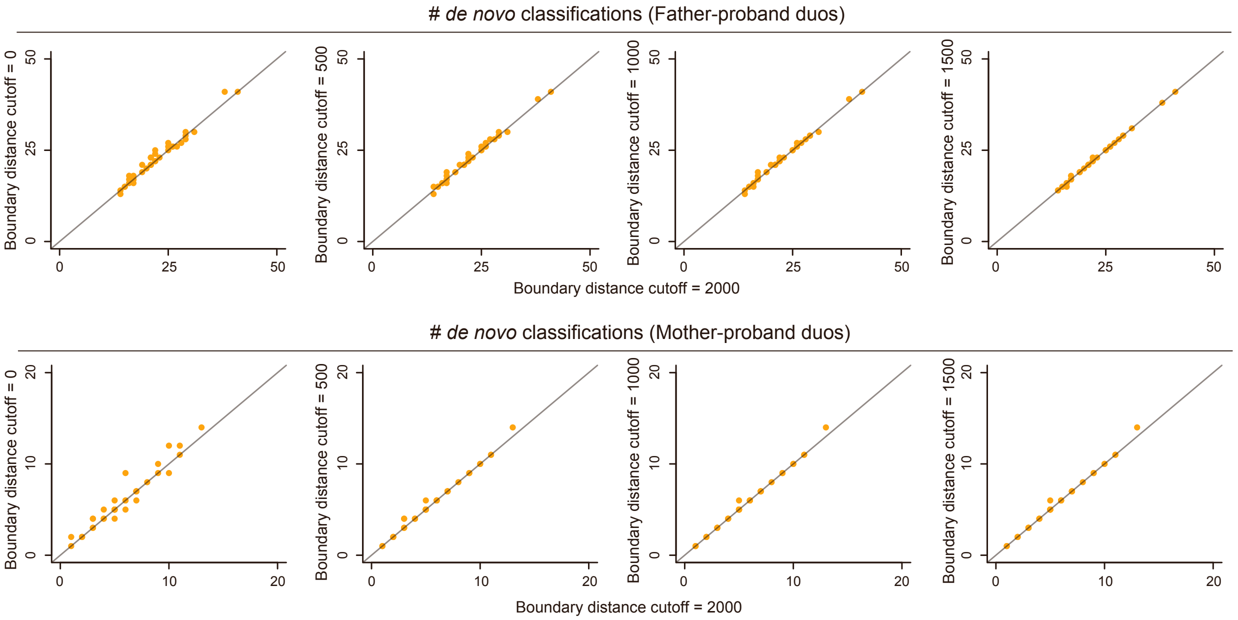

Figure S17. The impact of the distance threshold from haplotype block boundaries on the number of *de novo* classifications. Like Figure S15, but showing the impact of the threshold for the distance from the haplotype block boundaries (start/end coordinates) on the number of *de novo* classifications.

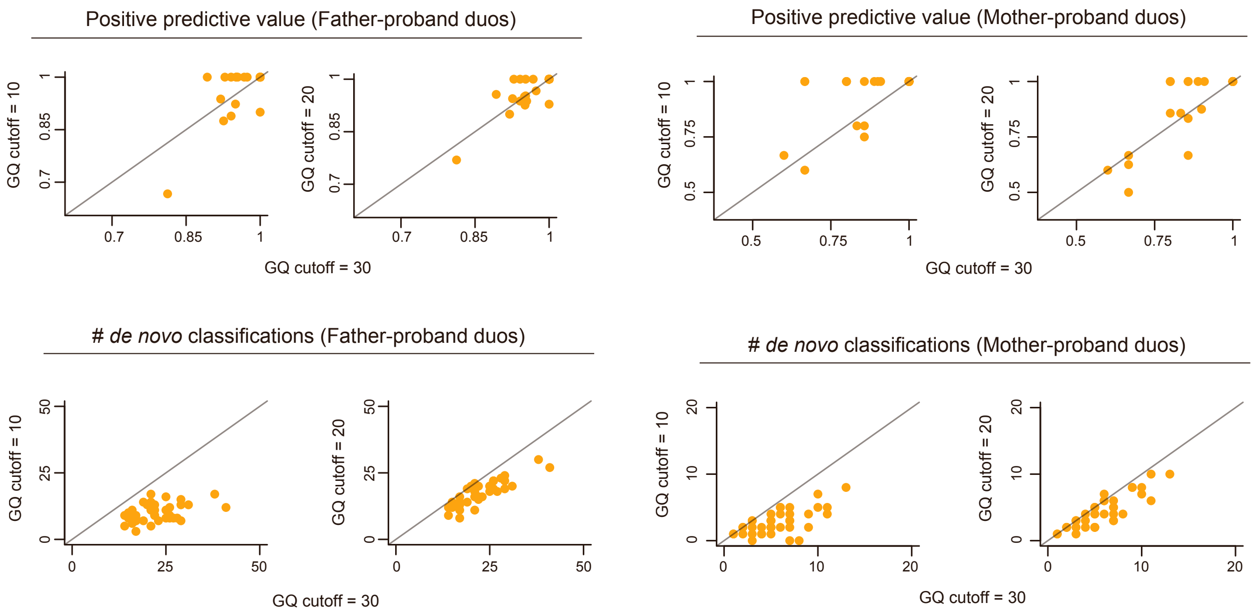

Figure S18. The impact of the phred quality (GQ) threshold at positions surrounding candidate variants on the positive predictive value and the number of *de novo* classifications. Like Figure S14 and Figure S15, but showing the impact of the GQ threshold for positions surrounding the candidate variant (that is, positions which determine the Hamming distance) on the PPV and the number of *de novo* classifications.

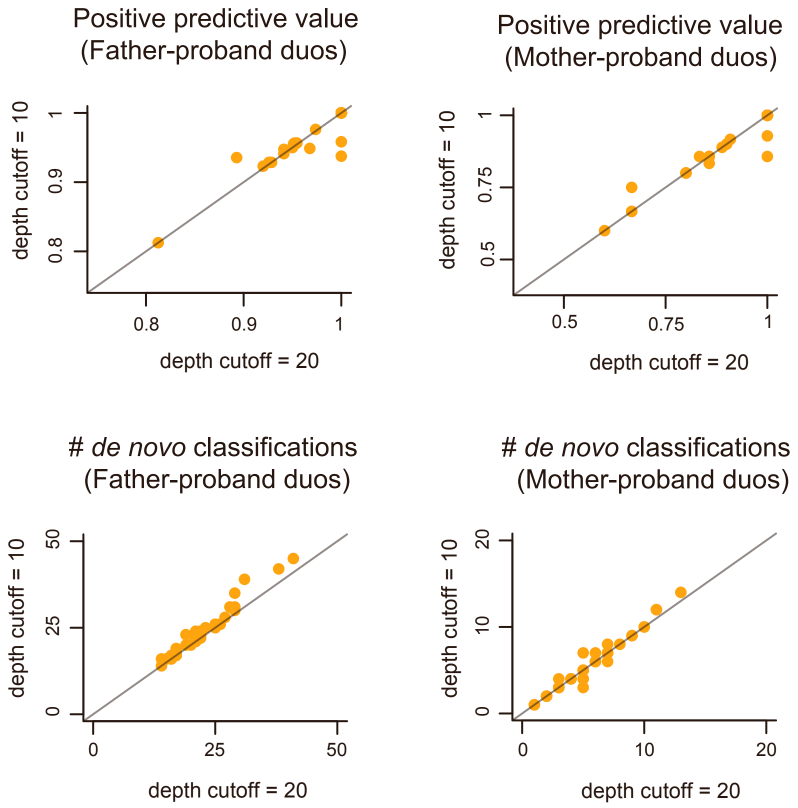

Figure S19. The impact of the sequencing depth threshold at positions surrounding candidate variants on the positive predictive value and the number of *de novo* classifications. Like Figure S18, but showing the impact of the sequencing depth threshold for positions surrounding the candidate variant (that is, positions which determine the Hamming distance) on the PPV and the number of *de novo* classifications.

Supplemental Tables

List of Supplemental Tables

1. **Table S 1 [Available as excel spreadsheet]**: Information (including ancestry, age of parents at childbirth, sequencing depth, and read length) of the participants comprising the 104 trios.

2. **Table S 2**: *duoNovo* Parameter thresholds for genome-scale vs targeted variant analysis (shown below).

3. **Table S 3 [Available as excel spreadsheet]**: List of variants classified as *de novo* by *duoNovo* from the 74 duos of the UCI-GREGoR case set. Includes ENSEMBL annotations based on location with respect to annotated genes, functional consequences for coding variants, variant frequency in different gnomAD populations, CADD Phred score, and spliceAI scores.

Table S 2. Parameter thresholds for genome-scale vs targeted variant analysis.

Parameter	Genome-scale analysis	Targeted variant analysis
Sequencing depth threshold	20	10
GQ threshold	30	20
Hamming distance criterion for hap. block similarity	$= 0$	≤ 2
Hamming distance criterion for hap. block dissimilarity	> 40	> 5
Distance from hap. block boundary threshold (bp)	2000	0
