# Supplementary material for: Identification of de novo variants from parent-proband duos via long-read sequencing
Source: Am J Hum Genet. 2026 Mar 5;113(3):437–52. doi: 10.1016/j.ajhg.2026.02.006 (PMC12987547; doi:10.1016/j.ajhg.2026.02.006)
Supplement: Document S2. Article plus supplemental information [file mmc4.pdf]

Identification of *de novo* variants from parent-proband duos via long-read sequencing

Graphical abstract

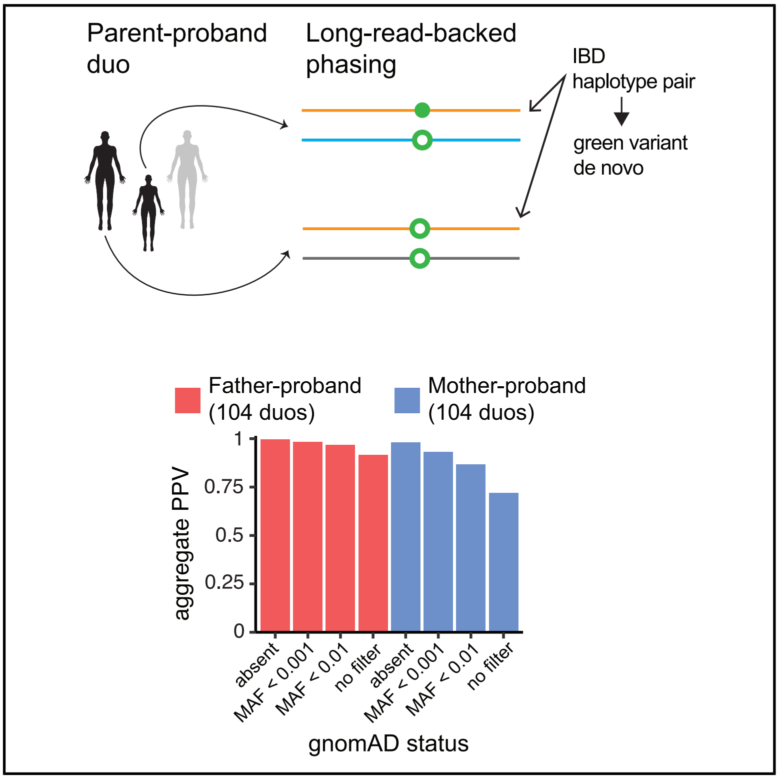

Authors

Leandros Boukas,
Emmanuèle C. Délot,
Georgia Pitsava, ..., Changrui Xiao,
Eric Vilain, Seth I. Berger

Correspondence

sberger@ambrygen.com

Determining *de novo* status is often what upgrades a variant from VUS to pathogenic, but it has not been possible without sequencing both biological parents. We developed, extensively evaluated, and applied a method that leverages long-read sequencing to detect *de novo* variants using only one biological parent.

Boukas et al., 2026, The American Journal of Human Genetics *113*, 437–452
March 5, 2026 © 2026 The Author(s). Published by Elsevier Inc. on behalf of American Society of Human Genetics.
https://doi.org/10.1016/j.ajhg.2026.02.006

CellPress

Identification of *de novo* variants from parent-proband duos via long-read sequencing

Leandros Boukas,[1,2,3] Emmanuèle C. Délot,[4] Georgia Pitsava,[4] Christine Lambert,[5] Cairbre Fanslow,[5] Primo Baybayan,[5] Sami Belhadj,[6] Bojan Losic,[6] John Harting,[6] Krista Bluske,[6] Jonathan LoTempio,[7] Huda B. Al-Kouatly,[8] Rachid Karam,[6] William J. Rowell,[5] Changrui Xiao,[4] Eric Vilain,[4] and Seth I. Berger[1,6,9,*]

Summary

While *de novo* variants cause many Mendelian disorders, their detection currently requires sequencing of the proband and both biological parents. This is not feasible when only one parent is available, a limitation for millions of families. Here, we develop *duoNovo*, which identifies *de novo* variants from parent-proband duos using long-read sequencing followed by haplotype reconstruction and detection of identical-by-descent haplotype blocks. We sequenced 104 trios with PacBio HiFi sequencing and applied *duoNovo* to each of the 208 duos constructed by masking one parent, classifying over 55 million variants according to their *de novo* status. We evaluated *duoNovo*'s performance against classifications obtained using the full trios (which included over 10,000 *de novo* variants), finding a positive predictive value of ∼98% among variants absent from gnomAD and a sensitivity of approximately 55% from father-proband duos (73% of the biological upper limit) and 14% from mother-proband duos (56% of the biological upper limit), the latter increasing to ∼24% when a sibling is available. In a cohort of 63 undiagnosed duos, *duoNovo* provided critical diagnostic information for two probands. In summary, *duoNovo* has the potential to significantly increase the diagnostic yield of single-parent genetic testing and represents an example where long-read sequencing provides a clear benefit over short-read sequencing even for single-nucleotide variants. It is freely available as an R package.

Introduction

De novo variants, which are present in affected probands but not (constitutively) present in the parents, underlie a substantial fraction of Mendelian disorders.[1–6] These disorders are typically highly penetrant and are often encountered as cases of severely affected probands with unaffected parents. Salient examples are developmental disorders; 76% of pathogenic variants identified in a recent large-scale study of approximately 3,600 trios were found to be *de novo*.[6]

The utility of *de novo* status in variant classification is further enhanced by the fact that *de novo* mutations are rare events, with ∼70 *de novo* mutations per genome per generation.[7,8] Knowing the *de novo* status of a variant thus aids significantly in variant prioritization. As a result, *de novo* status is considered moderate to strong evidence of variant pathogenicity under the guidelines of the American College of Medical Genetics and the Association for Molecular Pathology (ACMG/AMP).[9] Accordingly, variants with unknown *de novo* status are more likely to be designated as variants of uncertain significance (VUSs).[9–11]

Currently, *de novo* status can only be determined through sequencing of the proband and both biological parents. However, various factors can prevent both parents from participating in genetic testing. US Census statistics indicate that there are approximately 10 million single-parent families in the US alone (predominantly single-mother families). Additional reasons that make it infeasible to obtain samples from both biological parents include active military duty, children conceived through sperm or egg donation, or parental death. Consequently, probands in these families face a higher risk of non-diagnostic results[9–11] and have a higher rate of VUSs. Given that ACMG guidelines advise against using VUSs in treatment plan determinations, this can lead to missed opportunities to benefit from precision treatment and management options. Moreover, the magnitude of the reduction in diagnostic yield due to the unavailability of parental samples correlates with ancestry, indicating the potential for further exacerbation of existing health disparities.[6]

Long-read sequencing (LRS) is emerging in the clinical setting, although higher cost and uncertainty regarding diagnostic benefit over short-read sequencing (SRS) currently limit its widespread adoption.[12] To characterize the potential diagnostic benefit, a large body of work has focused on the ability of LRS to detect large/complex structural variants and other variants missed by SRS[13–20]; an additional studied benefit is the concurrent detection of epigenetic variation.[21] However, recent improvements

[1]Children's National Hospital, Washington, DC, USA; [2]Harvard Medical School, Boston, MA, USA; [3]Boston Children's Hospital, Boston, MA, USA; [4]University of California, Irvine, Irvine, CA, USA; [5]PacBio, Menlo Park, CA, USA; [6]Ambry Genetics, Aliso Viejo, CA, USA; [7]University of Pennsylvania, Philadelphia, PA, USA; [8]Thomas Jefferson University, Philadelphia, PA, USA; [9]The George Washington University, Washington, DC, USA
*Correspondence: sberger@ambrygen.com
https://doi.org/10.1016/j.ajhg.2026.02.006.
© 2026 The Author(s). Published by Elsevier Inc. on behalf of American Society of Human Genetics.
This is an open access article under the CC BY license (http://creativecommons.org/licenses/by/4.0/).

in LRS technologies, such as the development of PacBio HiFi LRS, now allow one to obtain reads with very high basecall accuracy,[18,22–26] enabling the identification of small variants, including single-nucleotide variants. One unique advantage of LRS is that it couples the identification of such variants with their reliable, read-backed phasing.[27,28] This is especially important for potentially disease-causing variants evaluated for *de novo* status in the clinical diagnostic setting, which are very rare or even unique to an affected individual and thus not amenable to phasing methods that rely on information about haplotype frequencies in the population.[29,30]

Here, we leverage these features of LRS to develop a method—which we call *duoNovo*—to identify *de novo* variants from duos (proband and one biological parent), with the potential to open more diagnostic opportunities for these families.

Methods

Participants

We sequenced 104 trios. 20 of these trios were derived from families with 2 siblings each, which were each split into two trios, whereas 3 trios were derived from a family with 3 siblings. Age (at childbirth) and genetic ancestry of the parents are provided in Table S1.

Ethics approval and consent to participate

All individuals consented to provide samples for genetic testing to the Pediatrics Mendelian Genomics Research Center (part of the GREGoR consortium). The study was approved by the Children's National Hospital institutional review board (IRB; IRB #PRO00015852).

LRS

Samples were prepared for LRS following the standard operating procedure (SOP) available at PACB.com ("preparing whole genome and metagenome libraries using SMRTbell prep kit 3.0"). Sequencing was performed on the PacBio Revio system with the Revio polymerase kit, following the Revio SMRT Link setup. Genomic DNA quality and concentration were assessed using the FEMTO Pulse (Agilent Technologies) and Qubit double-stranded DNA (dsDNA) high sensitivity (HS) reagents assay kit (Thermo Fisher Scientific). SMRTbell libraries were constructed on the Hamilton Microlab Star and VENUS 5 system following the same SOP. For library characterization, post-size-selected SMRTbell Library samples were quantified using the Qubit DNA HS assay, and DNA size was estimated using the FEMTO Pulse. The libraries were loaded at an on-plate concentration of 90 pM.

Raw sequencing reads were subsequently processed as follows. Circular consensus sequences were generated from subreads using CCS (v.7.0.0) with default settings, yielding demultiplexed HiFi reads. These reads were then aligned to the hg38 reference genome (https://github.com/PacificBiosciences/reference_genomes/blob/main/reference_genomes/human_GRCh38_no_alt_analysis_set/README.md) using PBMM2 (v.1.10.0), with default parameters.

Variant calling

Variant calling following LRS was performed using DeepVariant (v.1.5.0).[31,32] For each duo as well as for each trio, GVCFs were first generated, and joint variant calls were subsequently produced for each duo and trio separately with GLnexus (v.1.4.1) with the preset "DeepVariant_unfiltered." All our analyses and numbers reported are based on candidate *de novo* variants that were called both from a duo and its corresponding trio and thus were candidates we could evaluate.

For the evaluation of *duoNovo*'s performance, *de novo* classifications were determined to be correct if the non-sequenced parent did not have the candidate variant allele at that position (that is, if the non-sequenced parent was either homozygous for a non-variant allele or heterozygous with two different non-variant alleles). Similarly, non-*de-novo* classifications were determined to be correct if the non-sequenced parent had the candidate variant allele at that position. For these evaluations, we used genotype calls from the non-sequenced parent at positions with a sequencing depth of at least 20 and a PHRED genotype quality (GQ) of at least 30 (or 40 for the corresponding image in Figure 2C).

To obtain *de novo* variants from the entire trio, we used the bcftools +trio-dnm2 plugin (naive model)[33] to identify sites with a violation of Mendelian inheritance indicative of a *de novo* event (heterozygous variant in the proband; homozygous reference call in both parents). Given that identifying the variants that are *de novo* is known to be challenging even with access to a full trio, we then further filtered these variants using stringent criteria for variant and region quality. Specifically, from the variants that dnm2 identified as *de novo*, we retained those with a minimum GQ of 30 in each sample, a minimum depth of 20 reads in each sample, and 0 reads supporting the alternate allele in parents. We excluded variants within regions annotated as problematic by the Genome-in-a-Bottle consortium[34]; multi-allelic variant sites were also excluded. This yielded a median of 59 *de novo* variants (single-nucleotide variants and insertions or deletions [indels]) across all trios (range: 33–84), which is consistent with prior studies.[8,35] We recognize that the stringency of our filters means we have probably missed a few true *de novo* variants. However, for our purposes (evaluating *duoNovo*'s performance), this is preferable to having a benchmark set contaminated by false *de novo* calls.

In the 13 additional trios used for the analysis depicted in Figure S9, the artifactual *de novo* calls were introduced via joint variant calling performed using a different aligner version for either one or both parents compared to the proband. Specifically, in 4 of these trios, the mother was aligned using v.1.16.99 of the pbmm2 aligner (instead of v.1.10.0), whereas in 4 other trios, the father was aligned using v.1.16.99 instead of v.1.10.0. In these 8 trios—with only one parent aligned with a different aligner version—the genotype-driven approach identified between 150 and 300 *de novo* variants (Figure S9). In 4 other trios, only the proband was aligned using v.1.16.99, whereas in 1 trio, both parents were aligned using v.1.16.99. In these 5 trios—with both parents aligned using a different aligner version—the genotype-driven approach identified between 500 and 650 *de novo* variants (Figure S9). Not using the same aligner version for all members of a trio introduces artifactual *de novo* calls, mainly because of how indels and variants in low-complexity regions are represented. The only other analysis for which we used these trios was our examination of the fraction of classified variants from father-mother swapped duos (Figure S8), in which we included one

father-mother duo in which the mother was sequenced using a different aligner version, as an additional check of robustness.

Phasing

Following LRS, phasing was conducted using HiPhase (v.1.4.0)[28] with default parameters on the joint-called VCF from each duo. HiPhase assigned each phased variant to a phase set. Variants in the same phase set had the same phase relative to one another, enabling the resolution of those variants into haplotypes.

Classification of candidate *de novo* variants with *duoNovo*

Harmonizing proband/parent haplotype blocks

Following phasing, we imported the VCF files containing the phased variant calls into R with the VariantAnnotation package.[36] We then only retained variant positions where the sequencing depth was at least 20 and the GQ was at least 30. As stated in the results, we defined candidate *de novo* variants as variants heterozygous in the proband and absent in the parent.

To obtain classifications for these candidate variants, *duoNovo* first defines haplotype blocks as genomic regions within which (1) proband variants are assigned to the same phase set and (2) parent variants are assigned to the same phase set. To achieve this, *duoNovo* harmonizes phase set boundaries between the proband and the parent. It first obtains all pairs (i, j) where phase set i in the proband overlaps phase set j in the parent. For each pair, it then creates a new genomic interval corresponding to the overlap, whose start coordinate is the maximum of the two start coordinates and whose end coordinate is the minimum of the two end coordinates. These start/end coordinates are defined based on variants that pass the aforementioned depth and QC thresholds.

duoNovo then evaluates candidate variants separately in each of the two phasing orientations. Depending on whether the phasing orientation is "0|1" or "1|0," it determines which proband haplotype must exhibit high similarity with one of the two parental haplotypes and which proband haplotype must be dissimilar to both parental haplotypes, as described below.

Generating classifications for candidate variants

duoNovo sequentially examines each of the proband haplotype blocks containing candidate *de novo* variants. Each of these haplotype blocks is compared to each of the two corresponding parental haplotype blocks using the Hamming distance to quantify sequence similarity. In addition, *duoNovo* performs the same comparisons for the other proband haplotype (not containing the candidate variant). After performing these comparisons, *duoNovo* produces classifications based on the following criteria.

(1) If the proband haplotype block containing the candidate variant is highly similar to only one of the two parental haplotype blocks while the other proband haplotype block is highly dissimilar to both parental haplotype blocks, the candidate variant is classified as *de novo* (see next section for the determination of sequence similarity and dissimilarity based on the Hamming distance).
(2) If the proband haplotype block containing the candidate variant is highly dissimilar to both parental haplotype blocks while the other proband haplotype block is highly similar to only one of the two parental haplotype blocks, the candidate variant is classified as present on the haplotype inherited from the non-sequenced parent.
(3) If neither of the two above conditions is satisfied (e.g., due to no proband-parent haplotype pair passing the criterion for high similarity), the candidate variant does not receive a classification and is labeled as uncertain.

Requiring high similarity between the proband haplotype block and only one of the two parental haplotype blocks means that regions with parental runs of homozygosity will cause candidate variants to be classified as uncertain. This serves to minimize the potential for erroneous variant classifications due to parental "pseudohomozygosity" in cases where true heterozygous parental variants either do not pass depth/GQ thresholds or are not phased and thus do not enter the Hamming distance calculation.

Identifying highly similar and highly dissimilar haplotype blocks using the Hamming distance

In all cases, the Hamming distance between a pair of haplotype blocks is calculated after representing each haplotype block as a binary string (with 0 for the reference allele and 1 for the variant allele) and excluding candidate variants in the same phasing orientation as the candidate variant being evaluated.

For high similarity, a proband haplotype block must have a Hamming distance of 0 with the corresponding parental haplotype block. This is the most stringent choice (indicating perfect sequence similarity) and is our default for genome-scale analysis.

For high dissimilarity, a proband haplotype block must have a Hamming distance greater than 40 with the corresponding parental haplotype block. In the section parameter sensitivity analysis, we examine in detail the impact of the specific Hamming distance threshold used to define dissimilarity and find that smaller thresholds yield a lower positive predictive value (PPV), while higher thresholds do not make an appreciable difference.

For targeted variant analysis (that is, when testing variants for *de novo* status after manual curation has already deemed them to be of interest), we consider it reasonable to use more relaxed thresholds for haplotype similarity and dissimilarity compared to the aforementioned thresholds used for genome-scale analysis. This is because variants that have already undergone manual curation have a higher prior probability of being *de novo*. The thresholds that we used for our targeted analysis of variants from the UCI-GREGoR case set compared with those for genome-scale analysis are shown in Table S2 (the same table also shows differences in other parameter thresholds).

Interpretation of a small Hamming distance between a proband-parent haplotype block pair

Intuitively, a small Hamming distance between a proband-parent haplotype block pair indicates that this haplotype is shared identical by descent. Beyond this simple intuition, the precise information provided by the Hamming distance can be better understood by considering the genotypes of the different positions that determine its value (Figure S12).

(1) Positions where the proband is heterozygous and the parent is homozygous: these positions provide direct information about haplotype transmission, as only one of the two proband haplotypes could have been transmitted from the sequenced parent.
(2) Positions where both proband and parent are heterozygous: when considered in isolation, these positions do not provide direct information about haplotype transmission, as each of the two proband haplotypes could be identical by descent with the corresponding parental haplotype. However, when considered in the context of the

haplotype inferred to have been transmitted from the sequenced parent based on 1 above, these positions serve as a quality control (QC) for the accuracy of the phasing in the region, in both the proband and the parent.

(3) Positions where the proband is homozygous and the parent is heterozygous: these positions indicate which of the two parental haplotypes was transmitted to the proband, although in isolation, they cannot distinguish which of the two proband haplotypes was transmitted from the sequenced parent. In addition, when considered in the context of the haplotype, together with the heterozygous-heterozygous positions (type 2 above), they identify the proband-parent haplotype pair that is identical by descent. If the proband haplotype in this pair is not the same as that inferred from heterozygous-homozygous positions (type 1 above) to have been transmitted from the sequenced parent, then this suggests problematic phasing and/or variant calling in the region.

Consequently, when the Hamming distance between a pair of proband-parent haplotype blocks is very small, we can infer that (1) the proband inherited this haplotype block from the sequenced parent and not from the other (non-sequenced) parent and (2) the phasing in the region, based on which we have determined which proband haplotype contains the candidate variant being evaluated for *de novo* status, is accurate. We note here that, without read-backed phasing, candidate variants would always be classified as inherited from the non-sequenced parent, as they are absent in the sequenced parent. This highlights the critical role of read-backed phasing and the importance of ensuring its accuracy before classifying a candidate variant.

QC filtering beyond sequencing depth and PHRED GQ

After harmonizing parent/proband haplotype blocks, *duoNovo* discards candidate variants that are either in haplotype blocks smaller than 10 kb or at the boundaries of the remaining blocks (2 kb from the start/end coordinates). These boundary variants are discarded because we found that when they are classified as *de novo*, they tend to be false positives, especially in mother-proband duos (see section parameter sensitivity analysis).

To minimize false positive classifications due to genotyping errors, from the resulting classifications, we exclude variants within regions stratified as problematic by the Genome-in-a-Bottle consortium.[34]

Finally, if two or more of the variants classified as *de novo* are present within the same haplotype block, *duoNovo* discards all *de novo* classifications in that block and labels these variants as present on a "multi-*de-novo* haplotype." While these could represent true *de novo* events, we have observed that they tend to be false positives (see section "examining sources of false positive classifications").

Variants that fail QC vs. variants that are classified as uncertain

The following candidate variants are excluded by *duoNovo*'s QC filters.

(1) Variants that do not pass sequencing depth and/or GQ thresholds. These thresholds are tunable parameters.
(2) Variants that cannot be resolved into a haplotype block because HiPhase did not assign them to a phasing set (in either the proband or the parent).
(3) Variants in haplotype blocks whose size does not exceed the minimum chosen threshold. This threshold is a tunable parameter.
(4) Variants too close to the boundaries of a haplotype block, based on the chosen threshold for the distance from the boundary. This threshold is a tunable parameter.
(5) Variants within problematic regions (for example, as annotated by Genome-in-a-Bottle [GIAB]). One can choose whether or not to supply a list of such problematic regions.

The first 4 categories are variants for which *duoNovo* does not generate a classification. The final category (variants in problematic regions) is labeled as variants that failed QC, but we also output the classification that would have been received had they not been within a problematic region.

Examining the impact of the different QC filters, we found that, among variants that failed QC, in both father-proband and mother-proband duos, an average of 27% did so because they were not phased into a haplotype block (Figure S13A). On average, 31% of variants (in both father-proband and mother-proband duos) were located within a GIAB problematic region and, regardless of that, also failed a different QC step, while 29% were located within a GIAB problematic region but would have otherwise received a classification (Figure S13A). In addition, 8% and 9% of variants (on average) failed QC due to low sequencing depth in father-proband and mother-proband duos, respectively (Figure S13A). In contrast, removing haplotype blocks that do not exceed 10 kb and trimming the boundaries of haplotype blocks only affected a small minority of variants (less than 3% in both types of duos; Figure S13A).

We also further examined the impact of phasing independently of the other QC filters. We observed that the haplotype blocks (harmonized phasing sets between proband and parent) span an average of 2.12 and 2.16 billion bases in father-proband and mother-proband duos, respectively (decreasing to 2.06 and 2.09 after filtering out haplotype blocks not exceeding 10 kb and trimming the boundaries by 2 kb) (Figure S13B). The average fraction of candidate variants that remain unphased when no other QC filters (such as sequencing depth) are applied is 17% in father-proband and 18% in mother-proband duos (Figure S13C). These results show that improvements in phasing will further improve *duoNovo*'s ability to classify candidate variants.

In contrast to variants that fail QC, candidate variants that get classified as uncertain are variants that passed all the above QC filters, but the corresponding haplotype pair comparisons did not satisfy the criteria for identification of similar and dissimilar haplotype blocks (based on the chosen Hamming distance thresholds, which are tunable parameters) in order for the variants to receive a classification as either *de novo* or present on the non-sequenced parent's haplotype.

Variants in sex chromosomes

While *duoNovo* does not handle variants in sex chromosomes in a special fashion, our QC filters, as well as the underlying haplotype-similarity-based approach, safeguard against misclassifications of such variants. For instance, artifactual heterozygous variant calls on the Y or on the X chromosome in males (e.g., due to cross-mapping artifacts) typically get low GQ values and are also unlikely to be phased, as phasing by HiPhase relies on a sufficient density of heterozygous variants. Consistent with this, we found that among all variants classified as *de novo* from duos with male probands, only one is in a sex chromosome (classified as *de novo* from the father-proband duo). That one variant is in fact located within the pseudo-autosomal region of the X chromosome (chrX:2347174), suggesting it is a true heterozygous

variant. In future work, we plan to more systematically explore whether running DeepVariant with haploid contigs and pseudo-autosomal region coordinates as input affects *duoNovo*'s performance.

Out of the four types of duos (male proband-mother, male proband-father, female proband-mother, and female proband-father), the one type in which *duoNovo* is not able to generate classifications is that of female proband-father duos. In that case, because the father's X chromosome does not have phase sets (outside the pseudo-autosomal region), the phase set harmonization step cannot take place. In contrast, in the case of female proband-mother duos, candidate variants on the X chromosome can be handled in the same manner as in autosomes.

Related to this, the presence of deletions can create apparent haploid regions. In parents, these appear as regions of homozygosity, leading to candidate variants being labeled as uncertain. In probands, such deletions preclude the identification of candidate variants on the homologous chromosome that does not harbor the deletion, again because of apparent homozygosity. In future versions of *duoNovo*, we plan to integrate structural variant calling and assess the degree to which it improves our classifications.

Variant classification using proband-sibling-parent trios

For our analysis of variants using proband-sibling-mother and proband-sibling-father trios, we first constructed these trios from the families with siblings. Each sibling pair was analyzed twice, alternating which sibling was designated as the proband. This is because the set of candidate variants that are evaluated for *de novo* status is proband specific (being derived from the corresponding proband-parent duo). There were two families with 3 siblings each, from which 3 different proband-sibling-mother and 3 different proband-sibling-father trios were constructed.

Using these proband-sibling-parent trios, we generated joint-called phased trio VCF files in an identical fashion to the regular proband-father-mother joint-called phased trio VCFs.

For variant classification, we first obtained haplotype blocks within which (1) proband variants are assigned to the same phasing set, (2) sibling variants are assigned to the same phasing set, and (3) parent variants are assigned to the same phasing set. We subsequently defined candidate variants (heterozygous variant call in the proband and homozygous reference call in both the sibling and the parent) and proceeded to classify them. Specifically, a candidate variant was classified as *de novo* on the non-sequenced parent's haplotype if all of the following were satisfied.

(1) The proband haplotype block containing the candidate variant had a Hamming distance of 0 with exactly one of the two sibling haplotype blocks.
(2) The proband haplotype block without the candidate variant had a Hamming distance of 0 with exactly one of the two parental haplotype blocks.
(3) The proband haplotype block containing the candidate variant had a Hamming distance greater than 40 with both parental haplotype blocks.
(4) The proband haplotype block without the candidate variant had a Hamming distance greater than 40 with both sibling haplotype blocks.

When assessing the gain in sensitivity by using siblings as surrogates for the non-sequenced parent (Figure 4A), we obtained candidate variants among those previously classified—from the corresponding duo—as either present on the haplotype inherited from the non-sequenced parent or uncertain. When assessing the genome-scale PPV of the sibling trio-based approach (Figure 4B), candidate variants were obtained from the entire genome regardless of their previous classification from the corresponding duo.

Parameter sensitivity analysis

We chose the first 78 duos sequenced (39 father-proband; 39 mother-proband) to examine the impact of *duoNovo*'s tuning parameters on the PPV and the number of *de novo* classifications.

First, we varied the threshold for the Hamming distance used to determine if a pair of proband-parent haplotype blocks is dissimilar and thus not shared identically by descent (our default being 40). We found that a threshold of 0 always yields more *de novo* classifications compared to our default (as expected, since it is more lenient) but almost always yields a lower PPV, indicating that these classifications are enriched for false positives (Figures S14 and S15). Generally, we observed a slight improvement when increasing thresholds up to 40 but no net gain when using thresholds above 40.

Second, we varied the distance threshold from the boundaries of haplotype blocks. By default, *duoNovo* excludes variants within 2 kb of the start/end coordinates of haplotype blocks. We found that using a more lenient threshold tends to yield more false positives, especially in mother-proband duos (potentially reflecting a drop in phasing accuracy at these boundaries; Figure S16). The impact on the number of *de novo* classifications is minimal, as expected given that this threshold affects the inclusion of a comparatively small number of variants (Figure S17).

We then varied the thresholds for GQ and sequencing depth. One would naturally expect that using more lenient thresholds for candidate variants themselves would lead to an inflation of false positives. However, the impact of these thresholds when focusing on the positions surrounding candidate variants—which determine the Hamming distance between each proband-parent haplotype block pair—is less obvious. We found that, while using more lenient GQ thresholds (10 or 20 instead of 30) appears to increase the PPV (Figure S18), this was associated with a smaller number of variants being classified as *de novo*. This is likely due to the fact that a lower GQ causes haplotype block pairs that, in reality, are identical by descent to have a Hamming distance greater than 0 due to sequencing errors, thus leading to missed *de novo* variants. Finally, a more lenient sequencing depth threshold (10 instead of 20) had a small overall impact (Figure S19).

Examining sources of false positive classifications

As described above, from the resulting classifications, *duoNovo* excludes *de novo* variants clustered in the same haplotype block and variants within regions annotated as problematic by the Genome-in-a-Bottle consortium.[34] This is because we found that including these variants leads to an increased rate of false positive *de novo* classifications. Specifically, after pooling counts across all father-proband duos, we found that including variants within Genome-in-a-Bottle problematic regions reduces the PPV from 91.7% to 78.1% in father-proband duos and from 71.9% to 56.7% in mother-proband duos. Including variants classified as *de novo* and clustered in the same haplotype block reduces the PPV to 68% in father-proband duos and to 44.1% in mother-proband duos.

Finally, we found that both of these sources of false positive classifications have a smaller impact when restricting to variants absent from gnomAD. In that case, including variants within Genome-in-a-Bottle problematic regions reduces the aggregate

PPV to 96.4% in father-proband duos and to 84.3% in mother-proband duos. Including variants classified as *de novo* that are clustered in the same haplotype block reduces the aggregate PPV to 98.3% in father-proband duos and to 94.1% in mother-proband duos.

Variant annotation

We annotated all variants using ANNOVAR.[37] For each variant, annotations included genomic compartments (e.g., exonic, intronic, and intergenic), CpG vs. non-CpG context, gnomAD v.4.1 allele frequencies, and whether the variant falls into a GIAB problematic region.[34]

Relatedness and genetic ancestry calculation

Across all pairs of samples, relatedness was calculated using the "relate" function from Somalier[38] with default parameters. Genetic ancestry for each sample was predicted using the Somalier "ancestry" function using labeled data from the 1000 Genomes Project.

Mutation types

We calculated the number of occurrences of the different types of single-nucleotide variants per duo with the MutationalPatterns R package.[39] For Figure S5, percentages were calculated after pooling the counts across all father-proband duos and all mother-proband duos. The mutation types tested for different probabilities of occurrence in the paternal vs. the maternal germline were C>A, C>G, C>T, T>A, T>C, and T>G. C>T variants were tested separately for variants within and outside the CpG context.

De novo *variant curation from UCI-GREGoR duos*

Variants classified as *de novo* from the UCI-GREGoR case-set duos were annotated with ANNOVAR (as described above) and filtered to only retain variants that were (1) absent from gnomAD, (2) not intergenic, and (3) within genes with known dominant disease associations in OMIM. Subsequently, we further restricted to variants with evidence of potential pathogenicity. These were variants that were either designated as pathogenic in ClinVar or had a CADD Phred score greater than 20 or a spliceAI[40,41] score greater than 0.2. As described in results, these filters narrowed the list of *de novo* variants down to 4 candidate variants for manual review.

Results

duoNovo: Overview of approach

duoNovo uses haplotype blocks consisting of phased single-nucleotide variants across hundreds of kilobases (methods) in order to determine whether a candidate variant arose on the haplotype inherited from the sequenced parent—and is thus a *de novo* variant—or not. It achieves this by evaluating sequence similarity between pairs of haplotype blocks (methods). Each pair consists of a proband haplotype block and a parental haplotype block. If the proband haplotype block containing the variant being tested for *de novo* status exhibits high sequence similarity with one of the two parental haplotype blocks, then these haplotype blocks are inferred to be identical by descent, which in turn implies that the candidate variant is *de novo* (Figures 1A and 1B). If, on the other hand, it is the other proband haplotype (the one not containing the candidate variant) that has a highly similar sequence with one of the two parental haplotype blocks, then *duoNovo* infers that the haplotype containing the candidate variant was inherited from the missing biological parent (Figures 1A and 1B). Although in this latter case, the candidate variant may still be *de novo*, it is not possible to determine this from the available duo. As is evident, *duoNovo* critically relies on the read-backed phasing enabled by long reads and cannot be implemented with SRS instead (Figure S1).

duoNovo has near-perfect accuracy among variants absent from gnomAD

To assess the performance of *duoNovo*, we sequenced 104 trios using PacBio HiFi LRS to an average depth of ~34-fold across all individuals (SD: 4.4), obtaining approximately 16.8-kb-long reads on average (SD: ~2 kb; methods).

We started by applying *duoNovo* to each of the 208 duos (104 father-proband duos and 104 mother-proband duos), treating either the mother or the father as the non-sequenced parent (Figure 1C). We subjected each candidate variant (heterozygous phased variant call in the proband; homozygous reference call in the parent) to strict QC based on attributes including sequencing depth and GQ.

To evaluate *duoNovo*'s performance, we first sought to mimic a realistic rare disease diagnostic setting, where putative pathogenic variants have been filtered based on their frequency in control population databases. To this end, we focused on candidate variants that are absent from gnomAD v.4.1[42] and are thus enriched for rare pathogenic alleles. To evaluate the accuracy of each classification, we examined the non-sequenced parental sequence. For each variant *duoNovo* classified as *de novo* from the duo, we determined that the classification is correct if the variant is absent in the non-sequenced parent (Figure 1C).

duoNovo achieved very high accuracy. Collectively, 1,892 variants were classified as *de novo* from father-proband duos, among a total of 1,974,613 candidate variants (single-nucleotide variants and indels). From mother-proband duos, among a total of 1,934,021 candidate variants, 473 variants were classified as *de novo*. The average (per duo) PPV is 99.7% in father-proband duos and 97.9% in mother-proband duos (Figure 2A). In both father-proband and mother-proband duos, the PPV is equally high when focusing specifically on variants within genes (exonic or intronic regions), which are the most likely to be clinically relevant (Figure 2B).

When applying a more stringent GQ threshold (GQ > 40 as opposed to >30 in all members of the trio), we found that almost all of the false positives disappear. Out of the 1,685 and 398 variants classified as *de novo* in father-proband and mother-proband duos, respectively, there are only two false positives (one in a father-proband and one in a mother-proband duo; Figure 2C). This suggests that many of the apparent false positive *de novo* classifications are likely genotyping errors.

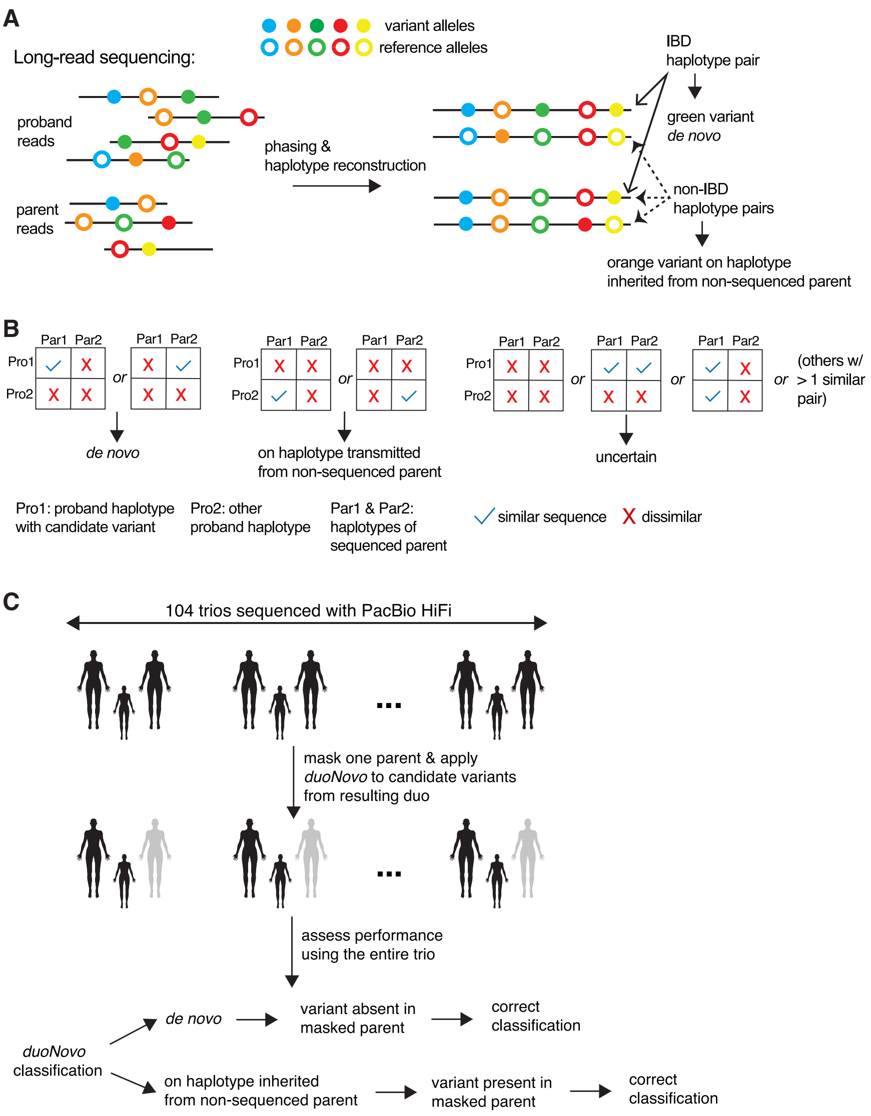

Figure 1. Detection of *de novo* variants from duos with *duoNovo*: Conceptual basis and overview of strategy for performance testing

(A) The long reads produced by PacBio HiFi sequencing (depicted by the black lines) enable the read-backed phasing of variants and the subsequent reconstruction of haplotypes. *duoNovo* leverages this to identify pairs of proband-parent haplotypes that are identical by descent based on their sequence similarity. It then classifies candidate variants (heterozygous in the proband; absent in the parent) on such identical-by-descent haplotypes as *de novo* (green variant in the cartoon figure; see also methods). On the other hand, candidate variants on haplotypes not identical by descent with any of the two parent haplotypes (orange variant in the cartoon figure) are inferred to have arisen on a haplotype inherited from the non-sequenced parent, in which case ascertaining their *de novo* status is not possible. Different positions are marked by different colors, and the variant allele at each position is depicted by the filled-in circle; the empty circles indicate the reference alleles at the corresponding positions.

(B) Different scenarios of proband-parent haplotype comparisons and the corresponding *duoNovo* classifications of the candidate variant (assumed to lie within proband haplotype 1).

(C) We sequenced 104 trios with PacBio HiFi sequencing (methods) and constructed father-proband and mother-proband duos by masking the mothers or the fathers, respectively. We then applied *duoNovo* to candidate variants from each duo, and the resulting variant classifications were evaluated based on the classifications that one would have obtained by having access to the entire trio.

duoNovo's PPV correlates with the stringency of gnomAD frequency and GQ filters

To gain further insight into *duoNovo*'s performance, we examined the accuracy of its *de novo* classifications among variants with different frequencies in gnomAD. The PPV decreases as the gnomAD frequency threshold becomes less stringent, as expected, given that the more common a variant is in the population, the higher its prior probability of being present in the non-sequenced parent (Figure 2C). When no frequency threshold is imposed, the aggregate PPV (computed after pooling classifications across all duos) is 91.7% in father-proband duos and 71.9% in mother-proband duos. Regardless of gnomAD frequency, we found that a more stringent GQ threshold leads to a higher PPV (Figure 2C). This is most pronounced when no allele frequency threshold is imposed, where the aggregate PPV increases from 91.7% (GQ $\geq$ 30) to 97.2% (GQ $\geq$ 40) in father-proband duos and from 71.9% (GQ $\geq$ 30) to 87.9% (GQ $\geq$ 40) in mother-proband duos, most likely due to the exclusion of genotyping errors.

In both father-proband and mother-proband duos, we observed no major difference in the PPV when the proband was of European vs. non-European ancestry (with slightly higher PPV in duos with non-European ancestry probands; Figure S2; methods).

duoNovo's classifications recapitulate the known age and parent of origin effects

When testing all variants that passed QC filters, regardless of gnomAD frequency, *duoNovo* classified 28 variants on average as *de novo* from father-proband duos (range: 12–51). Reassuringly, the number of *de novo* classifications positively correlates with father's age (Figure S3; Poisson regression $p = 1.29 \times 10^{-8}$). In mother-proband duos, *duoNovo* classified an average of 9 variants as *de novo*. With the exception of one trio, *duoNovo* always detected more *de novo* variants in father-proband duos compared to mother-proband duos, with the median ratio of paternally to maternally derived *de novo* classified variants approximately equal to 3 (Figure S4). This is consistent with the

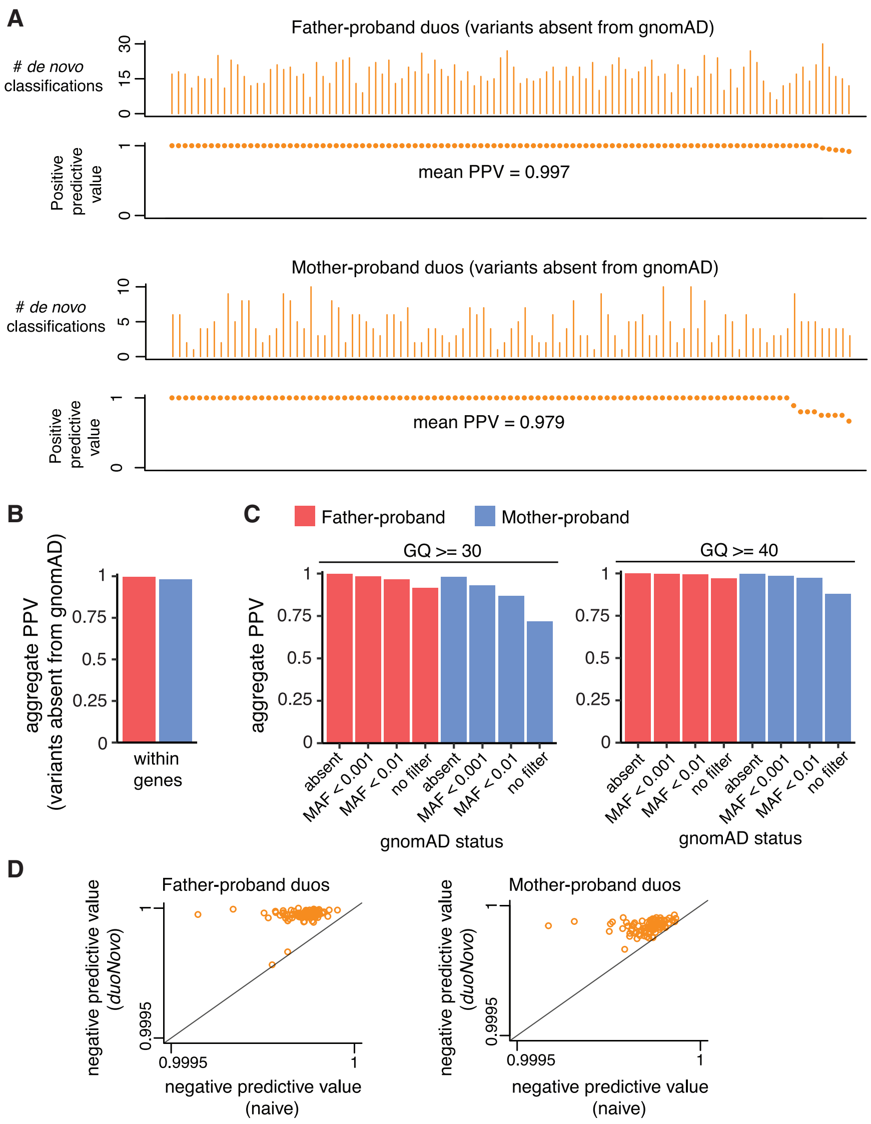

Figure 2. *duoNovo* has high positive and negative predictive values

(A) The positive predictive value (PPV) of *duoNovo* (bottom, y axis) and the number of variants classified as *de novo* (top, y axis). Both are calculated after first restricting to candidate variants absent from gnomAD (v.4.1). Each point and its associated vertical bar above corresponds to a duo. Duos are ordered on the x axis in decreasing PPV, and thus the order is different between the father-proband and mother-proband duos. In 5 mother-proband duos, there were no *de novo* classifications among variants absent from gnomAD; therefore, these duos are not visualized.

(B) Aggregate (across all duos) PPV, calculated among all candidate variants absent from gnomAD within genes (exons and introns).

(C) Aggregate (across all duos) PPV, calculated among all candidate variants after applying different thresholds to the gnomAD minor-allele frequency (MAF; x axis). Left figure shows aggregate PPV using a genotype quality threshhold of 30, and right figure shows the aggreggate PPV using a more stingent genotype quality threshhold of 40. In both cases, quality threshhold was applied to the evaluated variant position in all members of the trio.

(D) Scatterplots comparing the negative predictive value (NPV) of *duoNovo* (x axis) to the NPV of the naive baseline approach (classifying every variant as non-*de novo*; y axis). *duoNovo* always has a higher NPV, as indicated by the position of the points relative to the $y = x$ line.

elevated contribution of the paternal germline to the pool of *de novo* variants and estimates in prior literature[7,8,35,43] and can also explain why the PPV in mother-proband duos is lower compared to that in father-proband duos (Figure 2C).

Variants classified as *de novo* fall into expected mutation subtypes

In agreement with previously reported mutational patterns among *de novo* variants,[44,45] we found that the single-nucleotide variants *duoNovo* classified as *de novo* are mostly C>T and T>C substitutions (Figure S5), with the C>T substitutions occurring both within and outside the CpG context. There was no statistically significant difference in the proportion of the 6 mutation types among the *de novo* classifications from father-proband duos (2,699 single-nucleotide variants), and those from mother-proband duos (919 single-nucleotide variants) (Bonferroni-adjusted $p > 0.1$ for all types; Figure S5).

duoNovo has a very high negative predictive value

The negative predictive value of any sensible approach to classifying variants as *de novo* is expected to be high because *de novo* variants are extremely rare. To provide a fair assessment, we chose to compare *duoNovo* to the "naive baseline" approach: classifying every variant as non-*de novo*. We found that *duoNovo* always has a higher negative predictive value (Figures 2D and S6).

duoNovo very rarely classifies reference alleles as *de novo*

Our results so far have focused on candidate alternative alleles (1|0 or 0|1 proband genotype; 0/0 parental genotype). As an additional check of *duoNovo*'s performance, we compared the percentage of candidate alternative alleles classified as *de novo* to the corresponding percentage for candidate reference alleles (1|0 or 0|1 proband genotype; 1/1 parental genotype). Collectively, alternative candidate alleles were ~35 times more likely to be classified as *de novo* compared to reference candidate alleles in

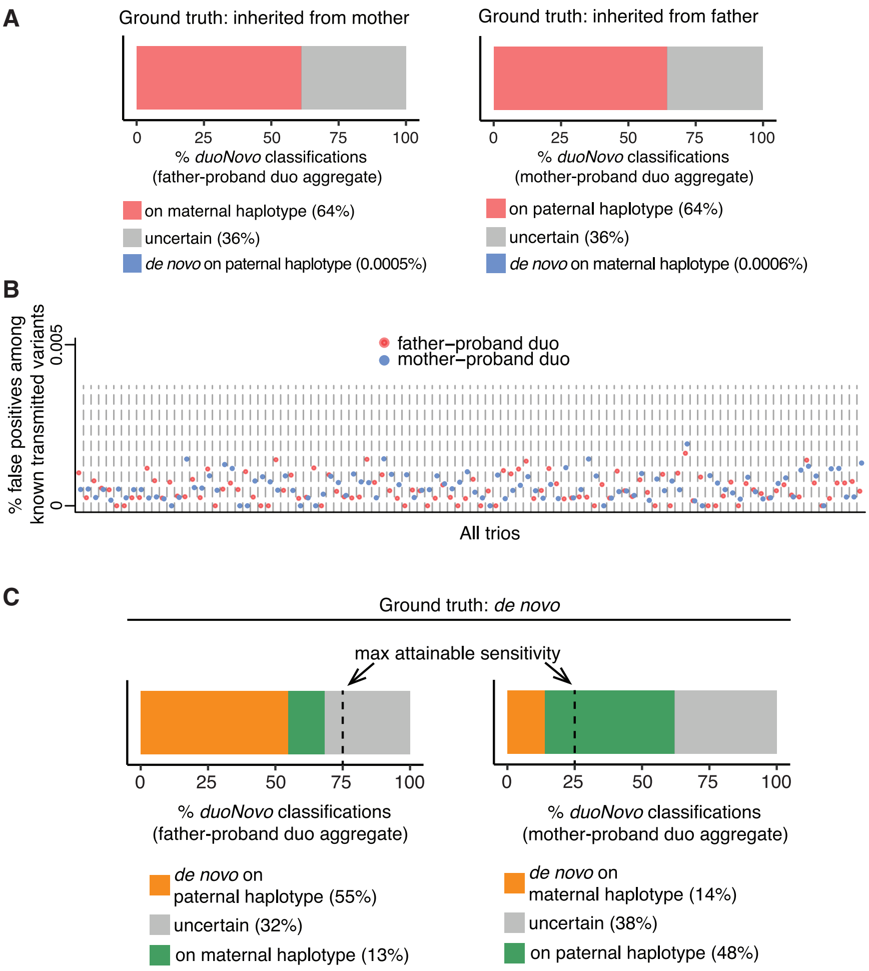

Figure 3. *duoNovo* has a very low error rate
(A) The percentage of candidate variants present in the non-sequenced parent (and thus inferred to be non-*de novo*) that are classified by *duoNovo* as present on the haplotype inherited from the non-sequenced parent, *de novo,* or uncertain. Percentages (*x* axis) were computed after pooling counts across all father-proband or mother-proband duos. Variants that did not pass QC filters required to proceed with classification (methods) or variants within a haplotype containing multiple *de novo* classifications (which are usually artifacts; methods) are excluded.
(B) The false positive rate (fraction of non-*de-novo* variants misclassified as *de novo*; *y* axis) of *duoNovo*, calculated separately for each father-proband and mother-proband duo.
(C) The percentage of *de novo* variants detected from the entire trio (methods) that were classified as *de novo*, present on the haplotype inherited from the non-sequenced parent, or uncertain. Percentages (*x* axis) were calculated after pooling counts across all father-proband or mother-proband duos. The dashed lines indicate the maximum attainable sensitivity from father-proband and mother-proband duos, based on the fact that approximately 75% and 25% of *de novo* variants are transmitted from the paternal and maternal germlines, respectively. Variants that failed QC are excluded from the percentage calculation.

father-proband duos and ∼12 times more likely in mother-proband duos. In 69 out of the 104 father-proband duos and 68 out of the 104 mother-proband duos, no reference alleles were classified as *de novo* (Figure S7). This further supports the accuracy of our *de novo* classifications, as the reference allele is common in the population and thus candidate reference alleles have a very low prior probability of being *de novo*.

duoNovo has a very low false positive rate

To directly quantify the genome-scale false positive rate of *duoNovo*, we subsequently restricted our attention to candidate variants that we inferred—based on the full trio information—to have been transmitted from the non-sequenced parent (reliable heterozygous phased variant call in the proband and reliable heterozygous or homozygous variant call in the non-sequenced parent; reliable homozygous reference call in the sequenced parent). We examined the fraction of these transmitted variants that are falsely classified as *de novo*. This fraction is defined as the false positive rate, which is equal to 1 − specificity, and is a distinct metric from the proportion of false *de novo* classifications among all *de novo* classifications, which is equal to 1 − PPV. We found that *duoNovo* has an average false positive rate across father-proband and mother-proband duos equal to 5.1×10^{-6} and 5.8×10^{-6}, respectively (Figures 3A and 3B). The majority (more than 60%) of these variants were correctly classified as present on the haplotype inherited from the non-sequenced parent, with almost 40% labeled as uncertain (Figure 3A). The maximum false positive rate across all duos was 1.9×10^{-5}.

As an orthogonal assessment of the false positive rate, we applied *duoNovo* to 40 swapped duos consisting of the father and the mother from the corresponding trio (that is, the proband was swapped with the parent previously treated as the non-sequenced one). From each swapped duo, we calculated the percentage of candidate variants that were classified as *de novo* or as present on the non-sequenced parent haplotype. Either classification requires the detection of a haplotype block shared identically by descent between the two members of the duo. We would thus expect the percentage of classified variants from these swapped duos to be very low, as fathers and mothers share a much smaller percentage of their genome identical by descent compared to father-proband or mother-proband pairs. Indeed, we found that the median percentage across swapped duos is 0.03%, compared to

65% across true parent-proband duos (Figure S8). One swapped duo stands out as an outlier, with 10.6% of variants receiving a classification (Figure S8); this is substantially higher than all the rest, though still well below the percentage from true parent-proband duos (Figure S8). Upon closer examination, we found that the father and mother from that swapped duo had the highest relatedness score among all father/mother pairs (methods; Figure S8), which could explain why *duoNovo* detected a larger fraction of identical-by-descent haplotype blocks.

Finally, we examined the number of variants classified as *de novo* by *duoNovo* from 13 additional trios that had multiple artifactual variant calls introduced during joint variant calling (methods). We compared it with the number of *de novo* variants detected using the standard approach, which consisted of identifying heterozygous variants in the proband (after stringent filtering) that were not present in either parent. We reasoned that since such artifactual variants should occur at random with respect to the surrounding haplotype context, *duoNovo* should not classify them as *de novo*, whereas the standard genotype-driven approach would be vulnerable and yield many false *de novo* calls. Indeed, we found that while the standard approach always yields unrealistically many *de novo* calls (354 on average; Figure S9), *duoNovo* never generates an excess of *de novo* classifications, and there is no correlation between the number of *de novo* calls using the genotype-driven approach and the number classified as *de novo* by *duoNovo* (Figure S9A; Pearson's $\rho = -0.15$, $p = 0.62$). This further supports the robustness of our haplotype-similarity-based approach. In contrast, in trios without such artifactual *de novo* calls, there is a strong positive correlation, as expected (Figure S9B; Pearson's $\rho = 0.75$).

duoNovo's sensitivity reflects the parent-of-origin effect

We next sought to characterize the sensitivity of *duoNovo*, based on a set of *de novo* variants detected using an established approach from the entire trio (hereafter referred to as trio-*de novo* variants; methods), which we treated as the ground truth. After excluding variants that failed QC, *duoNovo* collectively classified ~55% of trio-*de novo* variants as *de novo* from the father-proband duos and only ~14% as *de novo* from the mother-proband duos (Figure 3C). Conversely, approximately 13% were classified as present on the maternally inherited haplotype from the father-proband duos, whereas about 48% were classified as present on the paternally inherited haplotype from the mother-proband duos (Figure 3C). 32% and 38% of variants were labeled as uncertain from the father-proband and mother-proband duos, respectively. We highlight here that *duoNovo*'s sensitivity is biologically constrained, as it can only detect *de novo* variants that occurred on the haplotype of the sequenced parent. Therefore, the predicted maximum attainable sensitivity by a perfect method with perfect variant calls and phasing is around 25% for mother-proband duos and 75% for father-proband duos (since ~75% of *de novo* variants arise in the paternal germline and ~25% in the maternal germline[43]; Figure 3C). Our present sensitivity is thus approximately 73% that of the biological upper limit for father-proband duos and 56% for mother-proband duos.

Looking at the total fraction of trio-*de novo* variants that are classified as *de novo* from either the father-proband or the mother-proband duo, we found that the average sensitivity across all trios is 51% (Figure S10). The remaining were all variants that were either labeled as uncertain or did not receive a classification due to not passing *duoNovo*'s QC filters; none of the trio-*de novo* variants are misclassified as non-*de novo*.

Using siblings as surrogates for missing fathers boosts *duoNovo*'s sensitivity

The aforementioned fact that most *de novo* variants are transmitted from the paternal germline raises a practical issue pertinent to the clinical application of *duoNovo*, as most single-parent families are single-mother families. To mitigate this, we reasoned that siblings—when available—can increase *duoNovo*'s sensitivity by serving as surrogates for the missing father[46] since they share 50% of their genome with the father. To test this, we focused on 29 mother-proband duos where siblings were available. We first obtained the variants that *duoNovo* classified from the mother-proband duos as either present on the haplotype inherited from the missing father or uncertain. We then tested these variants for *de novo* status from the proband-sibling-mother trio (methods). We discovered that this leads to an 86% increase in sensitivity, from 12.8% to 23.9% (Figure 4A). This shows that the inclusion of siblings in the analysis can indeed partially compensate for the missing biological father. By contrast, and consistent with expectation, applying the same approach to proband-sibling-father trios only led to a negligible increase in the sensitivity compared to father-proband duos (57.3% compared to 54.6% without siblings; Figure 4A).

We also examined the genome-scale PPV of this approach. We obtained candidate variants based on genotype (heterozygous variant call in the proband; homozygous reference call in both the sibling and the parent), regardless of how these variants were classified from the proband-mother or proband-father duos. We classified these candidate variants directly using the corresponding proband-sibling-mother or proband-sibling-father trio and then examined the non-sequenced parent's genotype at the positions of *de novo* classifications to determine their correctness. We found that the PPV is equal to 72.3% and 74% from proband-sibling-mother and proband-sibling-father trios, respectively (Figure 4B).

duoNovo makes no erroneous classifications among a set of manually curated, clinically relevant variants

We next assessed *duoNovo*'s performance on a set of variants with known inheritance status that were manually curated

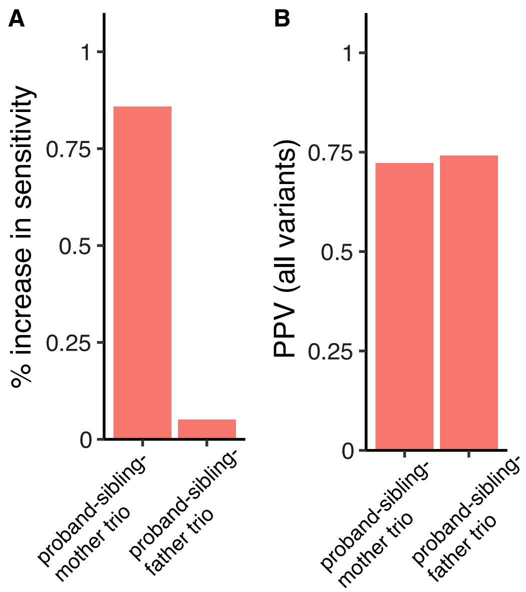

Figure 4. Using siblings as surrogates for the missing father increases the sensitivity of *duoNovo* from proband-mother duos
(A) The percentage of increase in sensitivity when using the proband-sibling-mother or proband-sibling-father trio to classify candidate variants initially classified as either present on the haplotype inherited from the non-sequenced parent, or uncertain, from the proband-mother or proband-father duo, respectively. Sensitivity is calculated after excluding variants that failed QC.
(B) The positive predictive value (PPV) when classifying candidate variants using either the proband-sibling-mother or the proband-sibling-father trio. The PPV is calculated here among candidate variants obtained from the entire genome (that is, without restricting to variants initially classified as either present on the haplotype inherited from the non-sequenced parent, or uncertain, from the duos).
Both (A) and (B) depict aggregate percentages, calculated after pooling classification counts across all 29 mother-proband and father-proband duos with siblings available (see methods for details).

as putatively clinically relevant in the UCI-GREGoR case set, a cohort of probands suspected to have a Mendelian disorder but who remained undiagnosed despite prior genetic testing.[47]

We first focused on 22 *de novo* variants, identified from 22 trios. We applied *duoNovo* to both the father-proband and the mother-proband duo corresponding to each trio, with settings adjusted for targeted variant analysis—which accounts for the higher prior probability of candidate variants deemed to be of clinical interest after manual curation as opposed to the lower prior of candidate variants identified genome wide solely based on genotype (methods). We classified 12 of the 22 variants as *de novo*, equivalent to a sensitivity of ~55% (Table 1). Unsurprisingly, 11 of the 12 *de novo* classifications came from the corresponding father-proband duo. Of the remaining variants, 5 were classified as on the non-sequenced parent haplotype from one duo but were either uncertain or failed QC (before proceeding to classification) from the other duo, 4 were classified as uncertain from both duos, and 1 was classified as uncertain from one duo but failed QC from the other duo. The predominant reason that variants failed QC in this analysis was that they were not resolved into a haplotype block for *duoNovo* to be able to classify them. Importantly, *duoNovo* did not misclassify any of these variants.

We also examined 41 non-*de-novo* variants for which we knew the parent of origin (17 paternal and 24 maternal). When applied to the duo consisting of the proband and the non-transmitting parent, *duoNovo* correctly classified 25 out of the 41 variants (61%) as present on the other parent's haplotype (Table 1). In all the remaining cases, the variant was either labeled as uncertain or failed QC; none of these variants were misclassified as *de novo*.

duoNovo provides critical diagnostic information for two probands among a cohort of undiagnosed rare disease cases

Finally, we applied *duoNovo* to 74 duos (52 mother-proband; 22 father-proband) from the UCI-GREGoR case set. Prior to applying *duoNovo*, 11 of these probands (from 3 father-proband and 8 mother-proband duos) had received a diagnosis after their enrollment in GREGoR, while the rest were still undiagnosed. Across all duos, *duoNovo* classified 917 variants as *de novo* (Table S3). After additional filtering to narrow this list down to variants likely to be clinically relevant (methods), we identified 4 candidate variants, three of which were previously reported as pathogenic in ClinVar. The first, identified in a mother-proband duo, is a pathogenic recurrent *de novo* variant in *RNU2-2* (n.4G>A [NCBI: NR_199791.1]), which causes a developmental and epileptic encephalopathy.[48,49] This variant—which explains the proband's phenotype—was previously missed because the disease association was not listed in OMIM at the time, but uncovering its *de novo* status by *duoNovo* led us to now reexamine the variant and deliver the diagnosis. The second variant is an insertion in *RNU4-2* (n.64_65insT [NCBI: NR_003137.3]) that was identified in a mother-proband duo where the proband had already received the diagnosis of ReNU syndrome. The maternal origin of the variant is consistent with the recent discovery that it is a recurrent *de novo* variant on the maternally transmitted haplotype in cases of neurodevelopmental delay.[50,51] The third *de novo* pathogenic variant—identified in a father-proband duo where the proband was also already diagnosed—is a variant in *ACTA1* (c.282C>A [GenBank: NM_001100.4] [p.Asn94Lys]), which explains the proband's congenital myopathy (MIM: 102610). In addition to these three pathogenic variants, we discovered a *de novo* intronic variant in *PHIP* (c.4206+3A>G [GenBank: NM_017934.7] [p.?]; Figure S11) in a proband with developmental delays and syndromic features. This variant, identified in a mother-proband duo, has a spliceAI score of 0.27, suggesting an impact on splicing.[41] Haploinsufficiency of *PHIP* causes Chung-Jansen syndrome (MIM: 617991), a disorder associated with developmental delays, intellectual

Table 1. **Performance of *duoNovo* on manually curated variants of clinical relevance from the UCI-GREGoR case set**

	Correct classification	Not classified	Incorrect classification
De novo	54.5% (12)	45.5% (10)	0% (0)
Maternally inherited	62.5% (15)	37.5% (9)	0% (0)
Paternally inherited	58.8% (10)	41.2% (7)	0% (0)

The *de novo* variants were tested from both duos. The maternally inherited variants were tested from the father-proband duo, while the paternally inherited variants were tested from the mother-proband duo.

disability, and distinct facial features.[52,53] While this variant is still classified as a VUS because of the broad phenotype, identification of its *de novo* status by *duoNovo* has made it a leading diagnostic candidate. Given that the proband's phenotype is consistent with Chung-Jansen syndrome, further functional studies are now planned to confirm the variant's pathogenicity. Taken together, these results show that *duoNovo* provided critical diagnostic information in 2 out of the 63 (3%) undiagnosed cases.

We also specifically looked at 10 variants that were already curated as putatively clinically relevant but were identified from duos and, as such, had uncertain *de novo* status. For these variants, we used the targeted *duoNovo* settings (methods). Of those variants, 8 were identified from mother-proband duos and 2 were identified from father-proband duos. After applying *duoNovo*, 7 of these variants were classified as present on the non-sequenced parent's haplotype (6 from mother-proband duos; 1 from a father-proband duo), 2 were uncertain, and one failed QC.

Discussion

We have developed and extensively evaluated a method that enables the identification of *de novo* variants from parent-proband duos. Our approach is simple and leverages the unique ability of LRS to both accurately detect and phase variants across large genomic segments.

Although LRS is currently more expensive than SRS, the cost differential between LRS and SRS has significantly decreased over the past decade,[54] and that trend is likely to continue as a result of continued improvements in technology. It is thus imperative to characterize the diagnostic benefit of LRS over SRS as accurately and comprehensively as possible. While an extensive body of work has established that LRS can detect variation that is undetectable with SRS (such as complex structural variants[13–20]), *duoNovo* illustrates that LRS can also enable the interpretation of variants that can be detected with SRS but are hard to interpret because of unknown *de novo* status. Importantly, the ability to ascertain *de novo* status from single-parent genetic testing promises to address an important source of inequity in result interpretation and diagnostic outcomes.

Conceptually, *duoNovo* is based on the recognition that phasing can provide information about haplotype transmission, and thus reveal variant inheritance, without access to both biological parent genotypes (see also Steyaert et al.[55]). An inherent limitation of our approach is that it can only detect *de novo* variants derived from the germline of the available sequenced parent. Given that the paternal germline contributes approximately 3–4 times as many *de novo* single-nucleotide variants as the maternal germline, whereas most single-parent families are single-mother families,[7,8,35] this will limit the yield in assigning *de novo* status to variants identified clinically. We show that this challenge can be partially addressed, however, if siblings are available and used as surrogates for the missing father. A potential source of false positives that one must be aware of when using the sibling trio-based approach is near-homozygous haplotypes in the non-sequenced parent, which differ only in the presence of the candidate variant. If the copy of the haplotype without the variant has been transmitted to the sibling, then the variant will be misclassified as *de novo* by the sibling-based trio approach.

An important question raised by our study is how *duoNovo* classifications can be incorporated into ACMG/AMP variant interpretation guidelines. We envision variants classified as *de novo* by *duoNovo* being treated equivalently to variants whose *de novo* status is asserted based on confirmed paternity and maternity, as *duoNovo*'s calls are based on inferring the local parentage at the level of the haplotype containing a variant. This is a major motivation behind design choices aimed at maximizing the accuracy of our classifications (prioritization of precision over recall, use of stringent variant quality thresholds, and use of the "uncertain" label as a possible output). Our results, especially among rare variants (which are the most relevant for the types of disorders usually caused by *de novo* variants), reflect this accuracy and provide fertile ground for further discussion by the expert review panels that develop these guidelines.

In this study, we have focused on single-nucleotide variants and indels. However, we anticipate that *duoNovo* is going to be similarly useful for additional variant types that LRS is capable of reliably detecting, such as structural variants and short tandem repeats.[56–61] Furthermore, although we used PacBio HiFi LRS to develop and test our method, *duoNovo* is generally applicable to any sequencing platform that generates accurate variant calls and enables read-backed haplotype reconstruction. Related to this, *duoNovo* is compatible with read-backed phasing methods other than HiPhase, such as WhatsHap,[27] though we currently recommend HiPhase, as it has been shown to have improved accuracy and can phase a larger portion of

the genome,[28] which enables a larger fraction of candidate variants to be classified with *duoNovo*.

Finally, we note that our current implementation of *duoNovo* uses certain hard thresholds for parameters relevant to variant call reliability, such as sequencing depth and GQ. These thresholds are supported by our extensive sensitivity analysis, although, as mentioned, we consider it sensible to use more lenient thresholds for targeted variant analyses as opposed to genome-scale analyses since the former focuses on variants that have already undergone manual curation and thus have a higher-than-baseline prior probability of being *de novo*. An alternative approach to the one we have taken here is to treat the thresholds as learnable parameters in a machine learning model trained to predict the *de novo* status of a variant. We view this as a very promising avenue for a future extension of *duoNovo*, though care must be taken when choosing the labeled examples to train the model on and to deal with issues related to class imbalance (*de novo* variants being very rare).

In summary, *duoNovo* is a systematic method that can identify *de novo* variants from duos with high accuracy at the genome scale. It has the potential to transform the diagnostic yield of genetic testing for millions of single-parent families and represents an example where LRS can provide a clear benefit compared to SRS in the clinical diagnostic setting. We anticipate that its implementation and free availability as an R package will facilitate its use and adoption by the community.

Data and code availability

Sequencing data are available on AnVIL as part of the GREGoR Consortium data release (https://gregorconsortium.org/data) with dbGaP: phs003047.v4. *duoNovo* is freely available for installation on https://github.com/sbergercnmc/duonovo. The v.1.0 release was used for this manuscript. The code used for analyses in this manuscript is available at https://github.com/sbergercnmc/duonovo/tree/main/paper_analysis_code.

Operating system(s): platform independent.
Programming language: R and Bash scripts.
Other requirements: VariantAnnotation R package.
License: MIT.
Any restrictions to use by non-academics: none.

Acknowledgments

Sample sequencing was performed through the UCI-GREGoR center, funded by NIH grant U01HG011745. L.B. was partly funded (until June 2025) through the Children's National Hospital Pediatric Residency Research, Education, and Advocacy for Children's Health (REACH) Program.

Author contributions

S.I.B. conceived of the study. L.B. and S.I.B. designed and implemented *duoNovo*. L.B. and S.I.B. processed and analyzed data. L.B. visualized the data. C.L., C.F., P.B., S.B., B.L., J.H., K.B., R.K., and W.J.R. performed the LRS and initial post-sequencing processing. L.B., G.P., and S.I.B. organized participant information. L.B. wrote the paper, which was then revised and edited by L.B. and S.I.B., with critical input from E.C.D., G.P., W.J.R., J.L., H.B.A.-K., C.X., and E.V. All authors reviewed the data and approved the final manuscript.

Declaration of interests

C.L., C.F., P.B., and W.J.R. are employees and shareholders of Pacific Biosciences. B.L., J.H., K.B., R.K., and S.I.B. are employees of Ambry Genetics.

Web resources

duoNovo R package github, https://github.com/sbergercnmc/duonovo
dbGaP, https://dbgap.ncbi.nlm.nih.gov/home/
GenBank, https://www.ncbi.nlm.nih.gov/genbank/
NCBI, https://static.pubmed.gov/portal/portal.fcgi/
OMIM, https://www.omim.org

Supplemental information

Supplemental information can be found online at https://doi.org/10.1016/j.ajhg.2026.02.006.

Received: October 5, 2025
Accepted: February 5, 2026
Published: March 5, 2026

References

1. Vissers, L.E.L.M., de Ligt, J., Gilissen, C., Janssen, I., Steehouwer, M., de Vries, P., van Lier, B., Arts, P., Wieskamp, N., del Rosario, M., et al. (2010). A de novo paradigm for mental retardation. Nat. Genet. *42*, 1109–1112. https://doi.org/10.1038/ng.712.
2. de Ligt, J., Willemsen, M.H., van Bon, B.W.M., Kleefstra, T., Yntema, H.G., Kroes, T., Vulto-van Silfhout, A.T., Koolen, D.A., de Vries, P., Gilissen, C., et al. (2012). Diagnostic exome sequencing in persons with severe intellectual disability. N. Engl. J. Med. *367*, 1921–1929. https://doi.org/10.1056/NEJMoa1206524.
3. Yang, Y., Muzny, D.M., Xia, F., Niu, Z., Person, R., Ding, Y., Ward, P., Braxton, A., Wang, M., Buhay, C., et al. (2014). Molecular findings among patients referred for clinical whole-exome sequencing. JAMA *312*, 1870–1879. https://doi.org/10.1001/jama.2014.14601.
4. Posey, J.E., Harel, T., Liu, P., Rosenfeld, J.A., James, R.A., Coban Akdemir, Z.H., Walkiewicz, M., Bi, W., Xiao, R., Ding, Y., et al. (2017). Resolution of disease phenotypes resulting from multilocus genomic variation. N. Engl. J. Med. *376*, 21–31. https://doi.org/10.1056/NEJMoa1516767.
5. Brunet, T., Jech, R., Brugger, M., Kovacs, R., Alhaddad, B., Leszinski, G., Riedhammer, K.M., Westphal, D.S., Mahle, I., Mayerhanser, K., et al. (2021). De novo variants in neurodevelopmental disorders-experiences from a tertiary care center. Clin. Genet. *100*, 14–28. https://doi.org/10.1111/cge.13946.

6. Wright, C.F., Campbell, P., Eberhardt, R.Y., Aitken, S., Perrett, D., Brent, S., Danecek, P., Gardner, E.J., Chundru, V.K., Lindsay, S.J., et al. (2023). Genomic diagnosis of rare pediatric disease in the United Kingdom and Ireland. N. Engl. J. Med. *388*, 1559–1571. https://doi.org/10.1056/NEJMoa2209046.
7. Jónsson, H., Sulem, P., Kehr, B., Kristmundsdottir, S., Zink, F., Hjartarson, E., Hardarson, M.T., Hjorleifsson, K.E., Eggertsson, H.P., Gudjonsson, S.A., et al. (2017). Parental influence on human germline de novo mutations in 1,548 trios from Iceland. Nature *549.7673*, 519–522. https://doi.org/10.1038/nature24018.
8. Sasani, T.A., Pedersen, B.S., Gao, Z., Baird, L., Przeworski, M., Jorde, L.B., and Quinlan, A.R. (2019). Large, three-generation human families reveal post-zygotic mosaicism and variability in germline mutation accumulation. eLife *8*, e46922. https://doi.org/10.7554/eLife.46922.
9. Richards, S., Aziz, N., Bale, S., Bick, D., Das, S., Gastier-Foster, J., Grody, W.W., Hegde, M., Lyon, E., Spector, E., et al. (2015). Standards and guidelines for the interpretation of sequence variants: a joint consensus recommendation of the American College of Medical Genetics and Genomics and the Association for Molecular Pathology. Genet. Med. *17*, 405–424. https://doi.org/10.1038/gim.2015.30.
10. Rehm, H.L., Alaimo, J.T., Aradhya, S., Bayrak-Toydemir, P., Best, H., Brandon, R., Buchan, J.G., Chao, E.C., Chen, E., Clifford, J., et al. (2023). The landscape of reported VUS in multigene panel and genomic testing: Time for a change. Genet. Med. *25*, 100947. https://doi.org/10.1016/j.gim.2023.100947.
11. Wojcik, M.H., Lemire, G., Berger, E., Zaki, M.S., Wissmann, M., Win, W., White, S.M., Weisburd, B., Wieczorek, D., Waddell, L.B., et al. (2024). Genome sequencing for diagnosing rare diseases. N. Engl. J. Med. *390*, 1985–1997. https://doi.org/10.1056/NEJMoa2314761.
12. Wojcik, M.H., Reuter, C.M., Marwaha, S., Mahmoud, M., Duyzend, M.H., Barseghyan, H., Yuan, B., Boone, P.M., Groopman, E.E., Délot, E.C., et al. (2023). Beyond the exome: What's next in diagnostic testing for Mendelian conditions. Am. J. Hum. Genet. *110*, 1229–1248. https://doi.org/10.1016/j.ajhg.2023.06.009.
13. Merker, J.D., Wenger, A.M., Sneddon, T., Grove, M., Zappala, Z., Fresard, L., Waggott, D., Utiramerur, S., Hou, Y., Smith, K.S., et al. (2018). Long-read genome sequencing identifies causal structural variation in a Mendelian disease. Genet. Med. *20*, 159–163. https://doi.org/10.1038/gim.2017.86.
14. Xie, Z., Sun, C., Zhang, S., Liu, Y., Yu, M., Zheng, Y., Meng, L., Acharya, A., Cornejo-Sanchez, D.M., Wang, G., et al. (2020). Long-read whole-genome sequencing for the genetic diagnosis of dystrophinopathies. Ann. Clin. Transl. Neurol. *7*, 2041–2046. https://doi.org/10.1002/acn3.51201.
15. Miller, D.E., Sulovari, A., Wang, T., Loucks, H., Hoekzema, K., Munson, K.M., Lewis, A.P., Fuerte, E.P.A., Paschal, C.R., Walsh, T., et al. (2021). Targeted long-read sequencing identifies missing disease-causing variation. Am. J. Hum. Genet. *108*, 1436–1449. https://doi.org/10.1016/j.ajhg.2021.06.006.
16. Mizuguchi, T., Okamoto, N., Yanagihara, K., Miyatake, S., Uchiyama, Y., Tsuchida, N., Hamanaka, K., Fujita, A., Miyake, N., and Matsumoto, N. (2021). Pathogenic 12-kb copy-neutral inversion in syndromic intellectual disability identified by high-fidelity long-read sequencing. Genomics *113*, 1044–1053. https://doi.org/10.1016/j.ygeno.2020.10.038.
17. Cohen, A.S.A., Farrow, E.G., Abdelmoity, A.T., Alaimo, J.T., Amudhavalli, S.M., Anderson, J.T., Bansal, L., Bartik, L., Baybayan, P., Belden, B., et al. (2022). Genomic answers for children: Dynamic analyses of > 1000 pediatric rare disease genomes. Genet. Med. *24*, 1336–1348. https://doi.org/10.1016/j.gim.2022.02.007.
18. Mastrorosa, F.K., Miller, D.E., and Eichler, E.E. (2023). Applications of long-read sequencing to Mendelian genetics. Genome Med. *15*, 42. https://doi.org/10.1186/s13073-023-01194-3.
19. Negi, S., Stenton, S.L., Berger, S.I., Canigiula, P., McNulty, B., Violich, I., Gardner, J., Hillaker, T., O'Rourke, S.M., O'Leary, M.C., et al. (2025). Advancing long-read nanopore genome assembly and accurate variant calling for rare disease detection. Am. J. Hum. Genet. *112*, 428–449. https://doi.org/10.1016/j.ajhg.2025.01.002.
20. Dominguez Gonzalez, C.A., Bell, K.M., Rajagopalan, R., de Silva, M.G., Lemes, A., Zabala, C., Pérez-Vidarte, F., Cerisola, A., Vossough, A., Whitehead, M.T., et al. (2025). Deep intronic SVA_E insertion identified as the most common pathogenic variant associated with Canavan disease: A diagnostic blind spot. Neurol. Genet. *11*, e200291. https://doi.org/10.1212/NXG.0000000000200291.
21. Vollger, M.R., Korlach, J., Eldred, K.C., Swanson, E., Underwood, J.G., Bohaczuk, S.C., Mao, Y., Cheng, Y.H.H., Ranchalis, J., Blue, E.E., et al. (2025). Synchronized long-read genome, methylome, epigenome and transcriptome profiling resolve a Mendelian condition. Nat. Genet. *57*, 469–479. https://doi.org/10.1038/s41588-024-02067-0.
22. Wenger, A.M., Peluso, P., Rowell, W.J., Chang, P.C., Hall, R.J., Concepcion, G.T., Ebler, J., Fungtammasan, A., Kolesnikov, A., Olson, N.D., et al. (2019). Accurate circular consensus long-read sequencing improves variant detection and assembly of a human genome. Nat. Biotechnol. *37*, 1155–1162. https://doi.org/10.1038/s41587-019-0217-9.
23. Kucuk, E., van der Sanden, B.P.G.H., O'Gorman, L., Kwint, M., Derks, R., Wenger, A.M., Lambert, C., Chakraborty, S., Baybayan, P., Rowell, W.J., et al. (2023). Comprehensive de novo mutation discovery with HiFi long-read sequencing. Genome Med. *15*, 34. https://doi.org/10.1186/s13073-023-01183-6.
24. Kolmogorov, M., Billingsley, K.J., Mastoras, M., Meredith, M., Monlong, J., Lorig-Roach, R., Asri, M., Alvarez Jerez, P., Malik, L., Dewan, R., et al. (2023). Scalable Nanopore sequencing of human genomes provides a comprehensive view of haplotype-resolved variation and methylation. Nat. Methods *20*, 1483–1492. https://doi.org/10.1038/s41592-023-01993-x.
25. Gustafson, J.A., Gibson, S.B., Damaraju, N., Zalusky, M.P., Hoekzema, K., Twesigomwe, D., Yang, L., Snead, A.A., Richmond, P.A., De Coster, W., et al. (2024). High-coverage nanopore sequencing of samples from the 1000 Genomes Project to build a comprehensive catalog of human genetic variation. In Genome Res., pp. gr.279273.124. https://doi.org/10.1101/gr.279273.124.
26. Hiatt, S.M., Lawlor, J.M.J., Handley, L.H., Latner, D.R., Bonnstetter, Z.T., Finnila, C.R., Thompson, M.L., Boston, L.B., Williams, M., Nunez, I.R., et al. (2024). Long-read genome sequencing and variant reanalysis increase diagnostic yield in neurodevelopmental disorders. In Genome Res. https://doi.org/10.1101/gr.279227.124.
27. Martin, M., Patterson, M., Garg, S., Fischer, S.O., Pisanti, N., Klau, G.W., Schöenhuth, A., and Marschall, T. (2016). WhatsHap: fast and accurate read-based phasing. bioRxiv. https://doi.org/10.1101/085050.

28. Holt, J.M., Saunders, C.T., Rowell, W.J., Kronenberg, Z., Wenger, A.M., and Eberle, M. (2024). HiPhase: jointly phasing small, structural, and tandem repeat variants from HiFi sequencing. Bioinformatics *40*, btae042. https://doi.org/10.1093/bioinformatics/btae042.
29. Browning, S.R., and Browning, B.L. (2011). Haplotype phasing: existing methods and new developments. Nat. Rev. Genet. *12*, 703–714. https://doi.org/10.1038/nrg3054.
30. Guo, M.H., Francioli, L.C., Stenton, S.L., Goodrich, J.K., Watts, N.A., Singer-Berk, M., Groopman, E., Darnowsky, P.W., Solomonson, M., Baxter, S., et al. (2024). Inferring compound heterozygosity from large-scale exome sequencing data. Nat. Genet. *56*, 152–161. https://doi.org/10.1038/s41588-023-01608-3.
31. Poplin, R., Chang, P.C., Alexander, D., Schwartz, S., Colthurst, T., Ku, A., Newburger, D., Dijamco, J., Nguyen, N., Afshar, P.T., et al. (2018). A universal SNP and small-indel variant caller using deep neural networks. Nat. Biotechnol. *36*, 983–987. https://doi.org/10.1038/nbt.4235.
32. Yun, T., Li, H., Chang, P.C., Lin, M.F., Carroll, A., and McLean, C.Y. (2021). Accurate, scalable cohort variant calls using DeepVariant and GLnexus. Bioinformatics *36*, 5582–5589. https://doi.org/10.1093/bioinformatics/btaa1081.
33. Danecek, P., Bonfield, J.K., Liddle, J., Marshall, J., Ohan, V., Pollard, M.O., Whitwham, A., Keane, T., McCarthy, S.A., Davies, R.M., and Li, H. (2021). Twelve years of SAMtools and BCFtools. GigaScience *10*, giab008. https://doi.org/10.1093/gigascience/giab008.
34. Dwarshuis, N., Kalra, D., McDaniel, J., Sanio, P., Alvarez Jerez, P., Jadhav, B., Huang, W.E., Mondal, R., Busby, B., Olson, N.D., et al. (2024). The GIAB genomic stratifications resource for human reference genomes. Nat. Commun. *15*, 9029. https://doi.org/10.1038/s41467-024-53260-y.
35. Kong, A., Frigge, M.L., Masson, G., Besenbacher, S., Sulem, P., Magnusson, G., Gudjonsson, S.A., Sigurdsson, A., Jonasdottir, A., Jonasdottir, A., et al. (2012). Rate of de novo mutations and the importance of father's age to disease risk. Nature *488*, 471–475. https://doi.org/10.1038/nature11396.
36. Obenchain, V., Lawrence, M., Carey, V., Gogarten, S., Shannon, P., and Morgan, M. (2014). VariantAnnotation: a Bioconductor package for exploration and annotation of genetic variants. Bioinformatics *30*, 2076–2078. https://doi.org/10.1093/bioinformatics/btu168.
37. Wang, K., Li, M., and Hakonarson, H. (2010). ANNOVAR: functional annotation of genetic variants from high-throughput sequencing data. Nucleic Acids Res. *38*, e164. https://doi.org/10.1093/nar/gkq603.
38. Pedersen, B.S., Bhetariya, P.J., Brown, J., Kravitz, S.N., Marth, G., Jensen, R.L., Bronner, M.P., Underhill, H.R., and Quinlan, A.R. (2020). Somalier: rapid relatedness estimation for cancer and germline studies using efficient genome sketches. Genome Med. *12*, 62. https://doi.org/10.1186/s13073-020-00761-2.
39. Manders, F., Brandsma, A.M., de Kanter, J., Verheul, M., Oka, R., van Roosmalen, M.J., van der Roest, B., van Hoeck, A., Cuppen, E., and van Boxtel, R. (2022). MutationalPatterns: the one stop shop for the analysis of mutational processes. BMC Genom. *23*, 134. https://doi.org/10.1186/s12864-022-08357-3.
40. Jaganathan, K., Kyriazopoulou Panagiotopoulou, S., McRae, J.F., Darbandi, S.F., Knowles, D., Li, Y.I., Kosmicki, J.A., Arbelaez, J., Cui, W., Schwartz, G.B., et al. (2019). Predicting splicing from primary sequence with deep learning. Cell *176*, 535–548.e24. https://doi.org/10.1016/j.cell.2018.12.015.
41. Walker, L.C., Hoya, M., Wiggins, G.A.R., Lindy, A., Vincent, L.M., Parsons, M.T., Canson, D.M., Bis-Brewer, D., Cass, A., Tchourbanov, A., et al. (2023). Using the ACMG/AMP framework to capture evidence related to predicted and observed impact on splicing: Recommendations from the ClinGen SVI Splicing Subgroup. Am. J. Hum. Genet. *110*, 1046–1067. https://doi.org/10.1016/j.ajhg.2023.06.002.
42. Karczewski, K.J., Francioli, L.C., Tiao, G., Cummings, B.B., Alföldi, J., Wang, Q., Collins, R.L., Laricchia, K.M., Ganna, A., Birnbaum, D.P., et al. (2020). The mutational constraint spectrum quantified from variation in 141,456 humans. Nature *581*, 434–443. https://doi.org/10.1038/s41586-020-2308-7.
43. Gao, Z., Moorjani, P., Sasani, T.A., Pedersen, B.S., Quinlan, A.R., Jorde, L.B., Amster, G., and Przeworski, M. (2019). Overlooked roles of DNA damage and maternal age in generating human germline mutations. Proc. Natl. Acad. Sci. USA *116*, 9491–9500. https://doi.org/10.1073/pnas.1901259116.
44. Kessler, M.D., Loesch, D.P., Perry, J.A., Heard-Costa, N.L., Taliun, D., Cade, B.E., Wang, H., Daya, M., Ziniti, J., Datta, S., et al. (2020). De novo mutations across 1,465 diverse genomes reveal mutational insights and reductions in the Amish founder population. Proc. Natl. Acad. Sci. USA *117*, 2560–2569. https://doi.org/10.1073/pnas.1902766117.
45. Shojaeisaadi, H., Schoenrock, A., Meier, M.J., Williams, A., Norris, J.M., Palmer, N.D., Yauk, C.L., and Marchetti, F. (2024). Mutational signature analyses in multi-child families reveal sources of age-related increases in human germline mutations. Commun. Biol. *7*, 1451. https://doi.org/10.1038/s42003-024-07140-2.
46. Young, C.L., Beichman, A.C., Mas-Ponte, D., Hemker, S.L., Zhu, L., Kitzman, J.O., Shirts, B.H., and Harris, K. (2024). A maternal germline mutator phenotype in a family affected by heritable colorectal cancer. In Genetics iyae166. https://doi.org/10.1093/genetics/iyae166.
47. Dawood, M., Heavner, B., Wheeler, M.M., Ungar, R.A., LoTempio, J., Wiel, L., Berger, S., Bernstein, J.A., Chong, J.X., Délot, E.C., et al. (2025). GREGoR: accelerating genomics for rare diseases. Nature *647*, 331–342. https://doi.org/10.1038/s41586-025-09613-8.
48. Greene, D., De Wispelaere, K., Lees, J., Codina-Solà, M., Jensson, B.O., Hales, E., Katrinecz, A., Nieto Molina, E., Pascoal, S., Pfundt, R., et al. (2025). Mutations in the small nuclear RNA gene RNU2-2 cause a severe neurodevelopmental disorder with prominent epilepsy. Nat. Genet. *57*, 1367–1373. https://doi.org/10.1038/s41588-025-02159-5.
49. Chiu, A.T.G., Bennett, M.F., Thiyagarajah, H., Schneider, A.L., Macdonald, S.M.W., Witkowski, T., Almanza Fuerte, E.P., Allan, T.J., Lieffering, N., Robinson, B., et al. (2025). Pathogenic variants in RNU2-2, a non-coding spliceosomal RNA, cause a distinctive developmental and epileptic encephalopathy. Ann. Neurol. 78071. https://doi.org/10.1002/ana.78071.
50. Chen, Y., Dawes, R., Kim, H.C., Ljungdahl, A., Stenton, S.L., Walker, S., Lord, J., Lemire, G., Martin-Geary, A.C., Ganesh, V.S., et al. (2024). De novo variants in the RNU4-2 snRNA cause a frequent neurodevelopmental syndrome. Nature *632*, 832–840. https://doi.org/10.1038/s41586-024-07773-7.
51. Greene, D., Thys, C., Berry, I.R., Jarvis, J., Ortibus, E., Mumford, A.D., Freson, K., and Turro, E. (2024). Mutations in the U4 snRNA gene RNU4-2 cause one of the most prevalent

monogenic neurodevelopmental disorders. Nat. Med. *30*, 2165–2169. https://doi.org/10.1038/s41591-024-03085-5.

52. Webster, E., Cho, M.T., Alexander, N., Desai, S., Naidu, S., Bekheirnia, M.R., Lewis, A., Retterer, K., Juusola, J., and Chung, W.K. (2016). De novo PHIP-predicted deleterious variants are associated with developmental delay, intellectual disability, obesity, and dysmorphic features. Cold Spring Harb. Mol. Case Stud. *2*, a001172. https://doi.org/10.1101/mcs.a00117.
53. Jansen, S., Hoischen, A., Coe, B.P., Carvill, G.L., Van Esch, H., Bosch, D.G.M., Andersen, U.A., Baker, C., Bauters, M., Bernier, R.A., et al. (2018). A genotype-first approach identifies an intellectual disability-overweight syndrome caused by PHIP haploinsufficiency. Eur. J. Hum. Genet. *26*, 54–63. https://doi.org/10.1038/s41431-017-0039-5.
54. McCombie, W.R., and McPherson, J.D. (2019). Future promises and concerns of ubiquitous next-generation sequencing. Cold Spring Harb. Perspect. Med. *9*, a025783. https://doi.org/10.1101/cshperspect.a025783.
55. Steyaert, W., Sagath, L., Demidov, G., Yépez, V.A., Esteve-Codina, A., Gagneur, J., Ellwanger, K., Derks, R., Weiss, M., den Ouden, A., et al. (2025). Unraveling undiagnosed rare disease cases by HiFi long-read genome sequencing. Genome Res *35*, 755–768. https://doi.org/10.1101/gr.279414.124.
56. Chaisson, M.J.P., Huddleston, J., Dennis, M.Y., Sudmant, P.H., Malig, M., Hormozdiari, F., Antonacci, F., Surti, U., Sandstrom, R., Boitano, M., et al. (2015). Resolving the complexity of the human genome using single-molecule sequencing. Nature *517*, 608–611. https://doi.org/10.1038/nature13907.
57. Sedlazeck, F.J., Rescheneder, P., Smolka, M., Fang, H., Nattestad, M., von Haeseler, A., and Schatz, M.C. (2018). Accurate detection of complex structural variations using single-molecule sequencing. Nat. Methods *15*, 461–468. https://doi.org/10.1038/s41592-018-0001-7.
58. Logsdon, G.A., Vollger, M.R., and Eichler, E.E. (2020). “Long-read human genome sequencing and its applications”. en. Nat. Rev. Genet. *21*, 597–614. https://doi.org/10.1038/s41576-020-0236-x.
59. Beyter, D., Ingimundardottir, H., Oddsson, A., Eggertsson, H.P., Bjornsson, E., Jonsson, H., Atlason, B.A., Kristmundsdottir, S., Mehringer, S., Hardarson, M.T., et al. (2021). Long-read sequencing of 3,622 Icelanders provides insight into the role of structural variants in human diseases and other traits. Nat. Genet. *53*, 779–786. https://doi.org/10.1038/s41588-021-00865-4.
60. Smolka, M., Paulin, L.F., Grochowski, C.M., Horner, D.W., Mahmoud, M., Behera, S., Kalef-Ezra, E., Gandhi, M., Hong, K., Pehlivan, D., et al. (2024). Detection of mosaic and population-level structural variants with Sniffles2. Nat. Biotechnol. *42*, 1571–1580. https://doi.org/10.1038/s41587-023-02024-y.
61. Figueroa, K.P., Gross, C., Buena-Atienza, E., Paul, S., Gandelman, M., Kakar, N., Sturm, M., Casadei, N., Admard, J., Park, J., et al. (2024). A GGC-repeat expansion in ZFHX3 encoding polyglycine causes spinocerebellar ataxia type 4 and impairs autophagy. Nat. Genet. *56*, 1080–1089. https://doi.org/10.1038/s41588-024-01719-5.

The American Journal of Human Genetics, Volume *113*

Supplemental information

Identification of *de novo* variants from parent-proband duos via long-read sequencing

Leandros Boukas, Emmanuèle C. Délot, Georgia Pitsava, Christine Lambert, Cairbre Fanslow, Primo Baybayan, Sami Belhadj, Bojan Losic, John Harting, Krista Bluske, Jonathan LoTempio, Huda B. Al-Kouatly, Rachid Karam, William J. Rowell, Changrui Xiao, Eric Vilain, and Seth I. Berger

Supplemental Figures

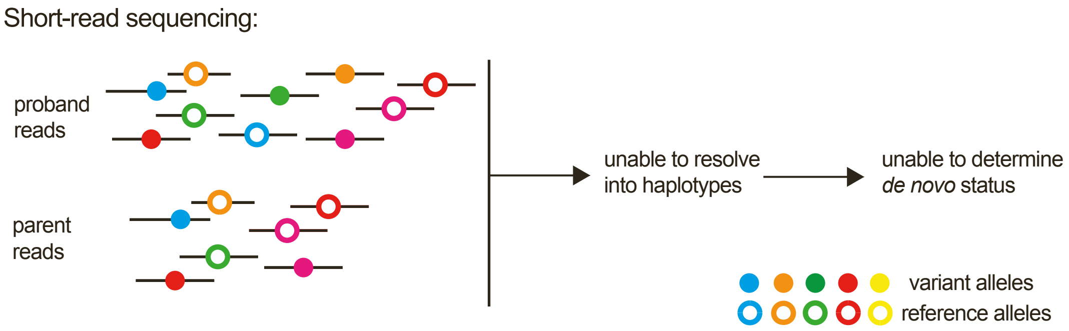

Figure S1. Cartoon representation of short reads. Like Figure 1A, but depicting short reads instead of long reads. Because of their short length, they (typically) do not contain more than one variant allele, thus precluding read-backed phasing and haplotype reconstruction.

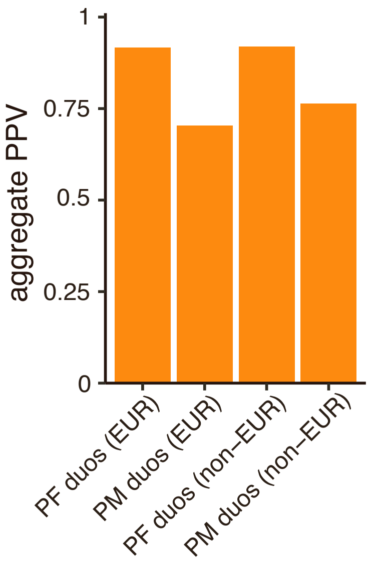

Figure S2. No major difference in the positive predictive value of *duoNovo* in probands of European vs non-European ancestry. Aggregate (across all duos) positive predictive value, shown separately for duos with probands of European versus non-European ancestry. Ancestry was calculated using Somalier[38]. Non-European ancestry groups include African (2 probands), Admixed American (17 probands), East Asian (3 probands), and South Asian (5 probands).

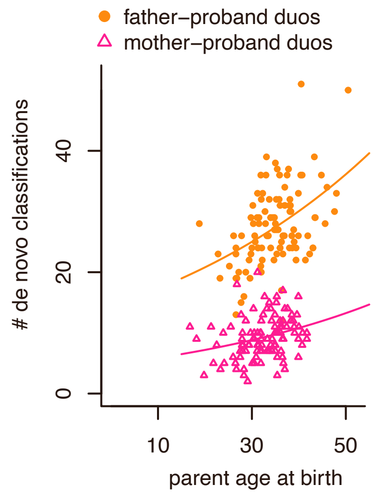

Figure S3. Paternal age positively correlates with the number of *de novo* classifications from father-proband duos. Each point corresponds to a duo. Trend lines were plotted after fitting Poisson regression models using the log link. As seen in previous studies[8], there is also a significant, but weaker, effect of maternal age.

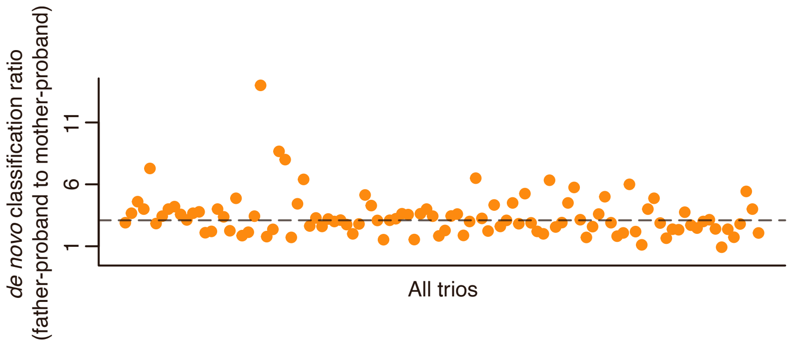

Figure S4. *duoNovo* detects more *de novo* variants from father-proband duos compared to mother-proband duos. Each point corresponds to a trio and its position on the y axis corresponds to the ratio of the number of *de novo* classifications from the father-proband duo to the number of *de novo* classifications from the mother-proband duo. The dashed horizontal line corresponds to the median across all trios.

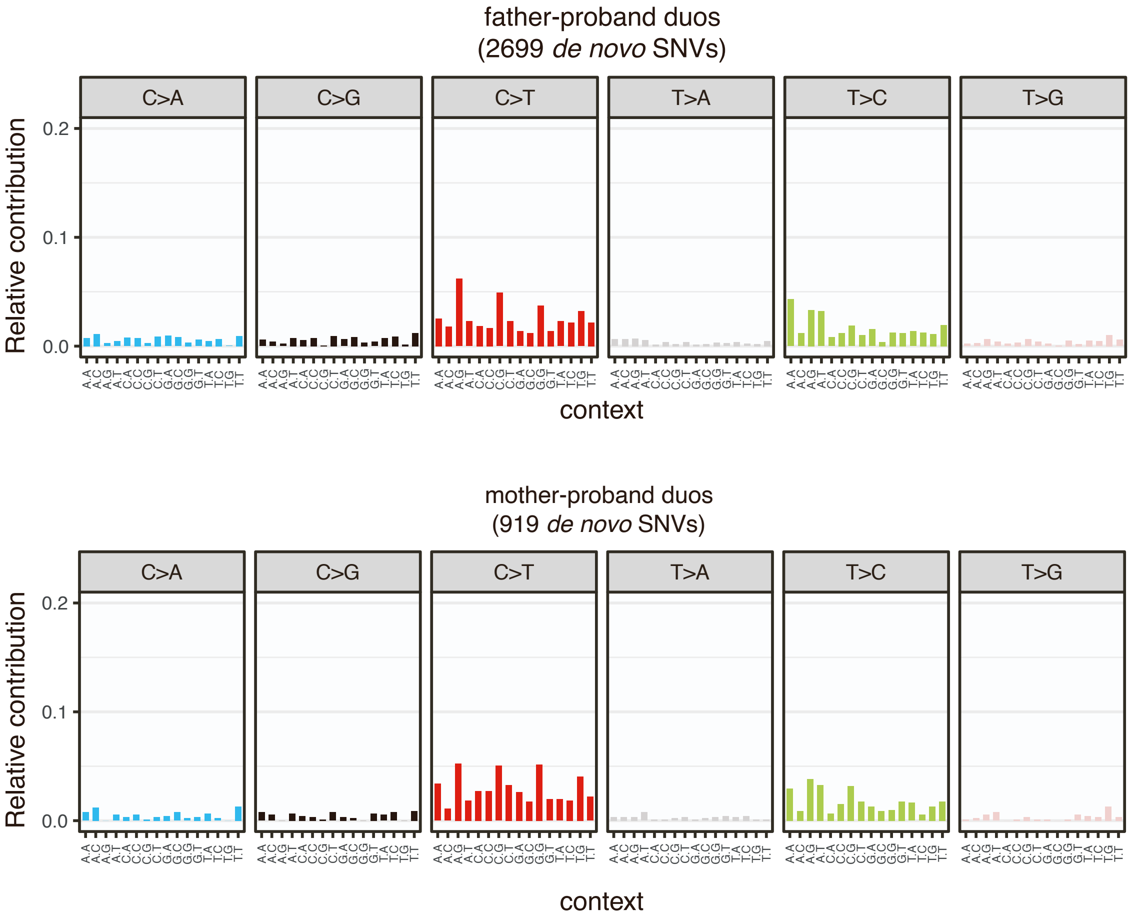

Figure S5. The *de novo* single nucleotide variants identified by *duoNovo* fall into expected mutation subtypes. The relative contributions were calculated after pooling all single nucleotide variants classified as *de novo* from the father-proband duos (top) and the mother-proband duos (bottom).

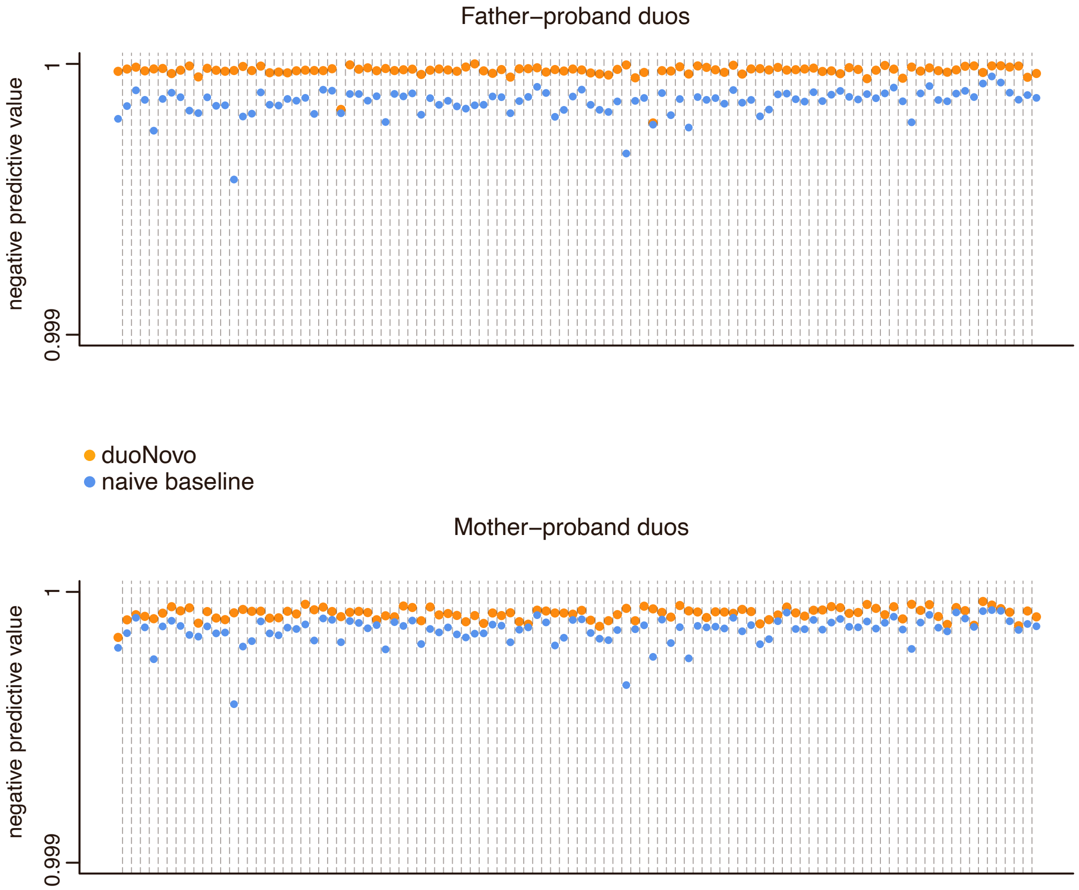

Figure S6. *duoNovo* has higher negative predictive value than the naive baseline (classifying every variant as non-*de novo*). The negative predictive value (y axis) of *duoNovo* (orange points) and of the naive baseline approach (blue points) for each father-proband and mother-proband duo.

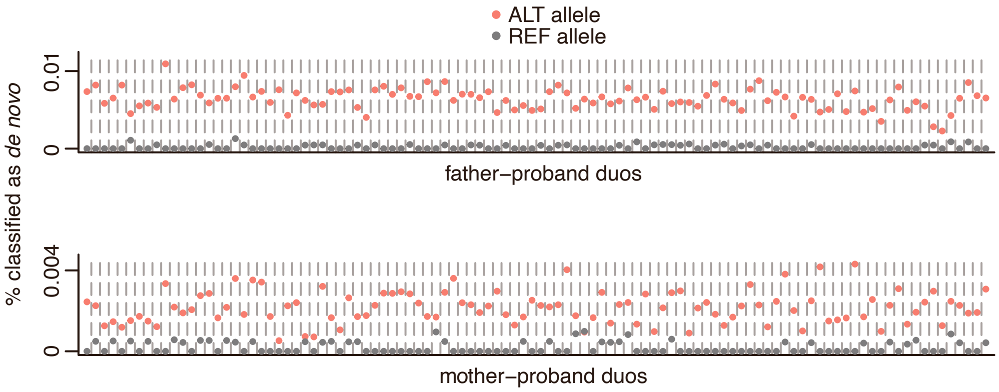

Figure S7. ***duoNovo* very rarely classifies candidate reference alleles as *de novo*.** Candidate alternative (ALT) alleles were identified by finding positions where the proband had the "1|0" or "0|1" genotype and the parent had the "0/0" genotype. Conversely, candidate reference (REF) alleles were identified by finding positions where the proband had the "1|0" or "0|1" genotype and the parent had the "1/1" genotype. Positions were either the proband or the parent failed QC filters (Methods) were excluded.

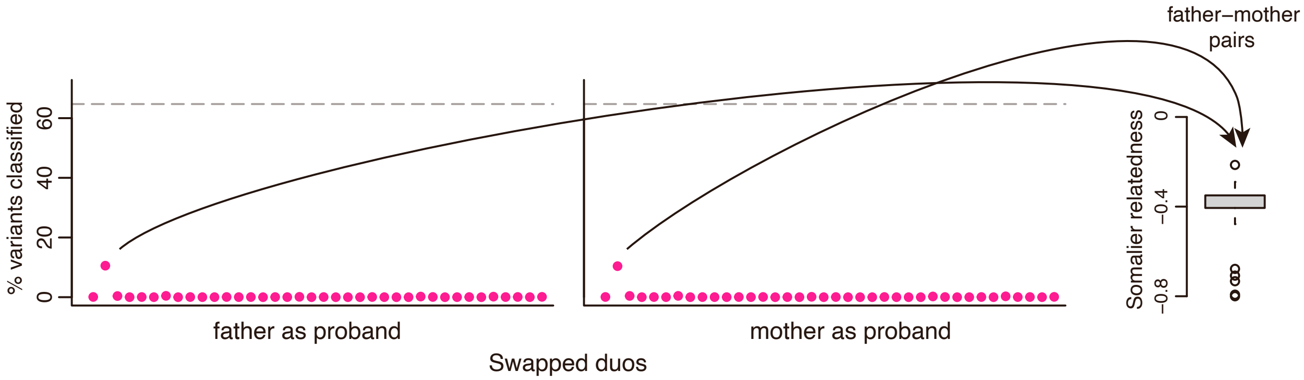

Figure S8. ***duoNovo* does not classify variants from swapped duos.** Each point corresponds to a swapped duo, and its position on the y axis indicates the percentage of variants that received a classification (either *de novo* or on the non-sequenced parent haplotype). Each swapped duo consists of the two parents instead of a parent and the proband; *duoNovo* was applied to each swapped duo by either treating the father as the proband (left) or the mother as the proband (right). The dashed horizontal line corresponds to the median percentage of variants that received a classification across all regular (father-proband or mother-proband) duos. The rightmost panel depicts the distribution of relatedness coefficients between all the fathers and mothers (each point corresponds to a father-mother pair), showing that the outlier swapped duo is the one with the highest relatedness coefficient.

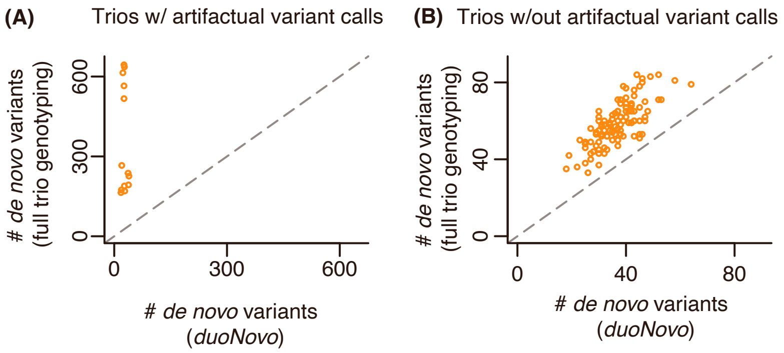

Figure S9. *duoNovo* does not classify artifactual candidate variants as *de novo*. (a) Each point corresponds to a trio with multiple artifactual variant calls generated during joint variant calling (Methods). The x-axis depicts the number of *duoNovo*'s *de novo* classifications after summing classifications from father-proband and mother-proband duos. The y-axis depicts the number of *de novo* variant calls using the standard genotype-driven approach. The dashed line is the $y = x$ line. **(b)** Like **(a)**, but for trios without artifactual variant calls. Unlike **(a)**, there is a strong correlation between the number of *de novo* variant calls using the standard genotype-driven approach and the number of total (summing father-proband and mother-proband duos) *de nono* classifications from *duoNovo*.

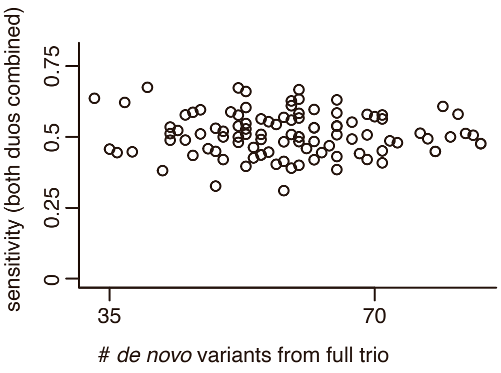

Figure S10. The relationship between *duoNovo*'s sensitivity (when combining *de novo* variants detected from father-proband and mother-proband duos) and the number of ground truth *de novo* variants detected using the standard genotype-driven approach from the full trio. Each point corresponds to a trio. The calculation of sensitivity includes trio *de novo* variants that did not receive a classification by *duoNovo* because they failed QC filtesr.

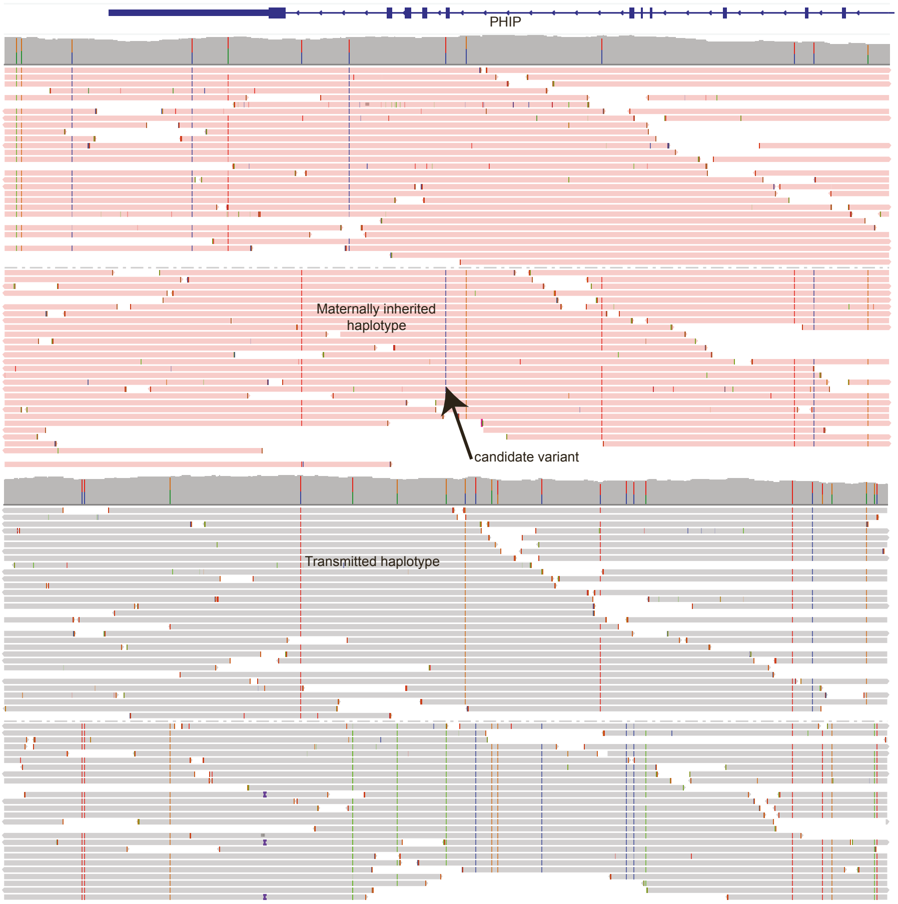

Figure S11. IGV snapshot of the sequencing reads from the proband harboring the *PHIP* intronic variant classified as *de novo* and the mother. Proband reads are shown in pink at the top and are grouped according to phase; the dashed line in the middle separates the two groups. Similarly, the maternal sequencing reads are shown in gray at the bottom grouped according to phase, with the dashed gray line separating the two groups.

Proband haplotypes

0 -- 1 -- 0 -- 1 -- 0 -- 0 -- 0 -- 1 -- 0 -- 0
0 -- 0 -- 1 -- 0 -- 1 -- 1 -- 1 -- 0 -- 1 -- 0

Parent haplotypes

0 -- 1 -- 0 -- 1 -- 0 -- 0 -- 0 -- 1 -- 0 -- 0
1 -- 0 -- 1 -- 1 -- 0 -- 0 -- 0 -- 0 -- 1 -- 1

Figure S12. Different types of genotypes that determine the Hamming distance between a proband and a parent haplotype. Red – positions heterozygous in the proband and homozygous in the parent. Blue – positions heterozygous in both proband and parent. Cyan – positions homozygous in the proband and heterozygous in the parent. The haplotypes are depicted as binary strings, with 1 indicating the variant allele and 0 the reference allele at each position.

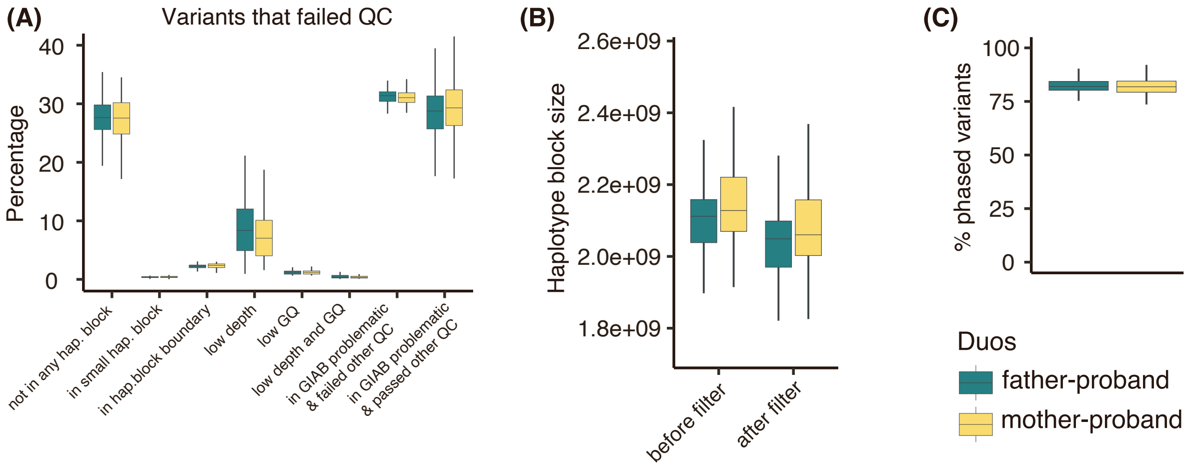

Figure S13. Lack of phasing and presence within a GIAB problematic region are the main reasons variants do not pass *duoNovo*'s QC steps. (a) Distributions (across father-proband and mother-proband duos) of the fraction of candidate variants that failed different QC steps. For each duo, each fraction is calculated among all variants that failed QC. Haplotype blocks refer to harmonized parent-proband phasing sets (Methods). **(b)** The distribution of the cumulative size of all haplotype blocks before and after applying the two relevant filters (removing haplotype blocks with size $\leq$ 10 kb and trimming the boundaries, i.e. 2 kb on either side). **(c)** The distribution of the total fraction of candidate variants that were phased across father-proband and mother-proband duos, irrespectively of other filters such as sequencing depth and GQ.

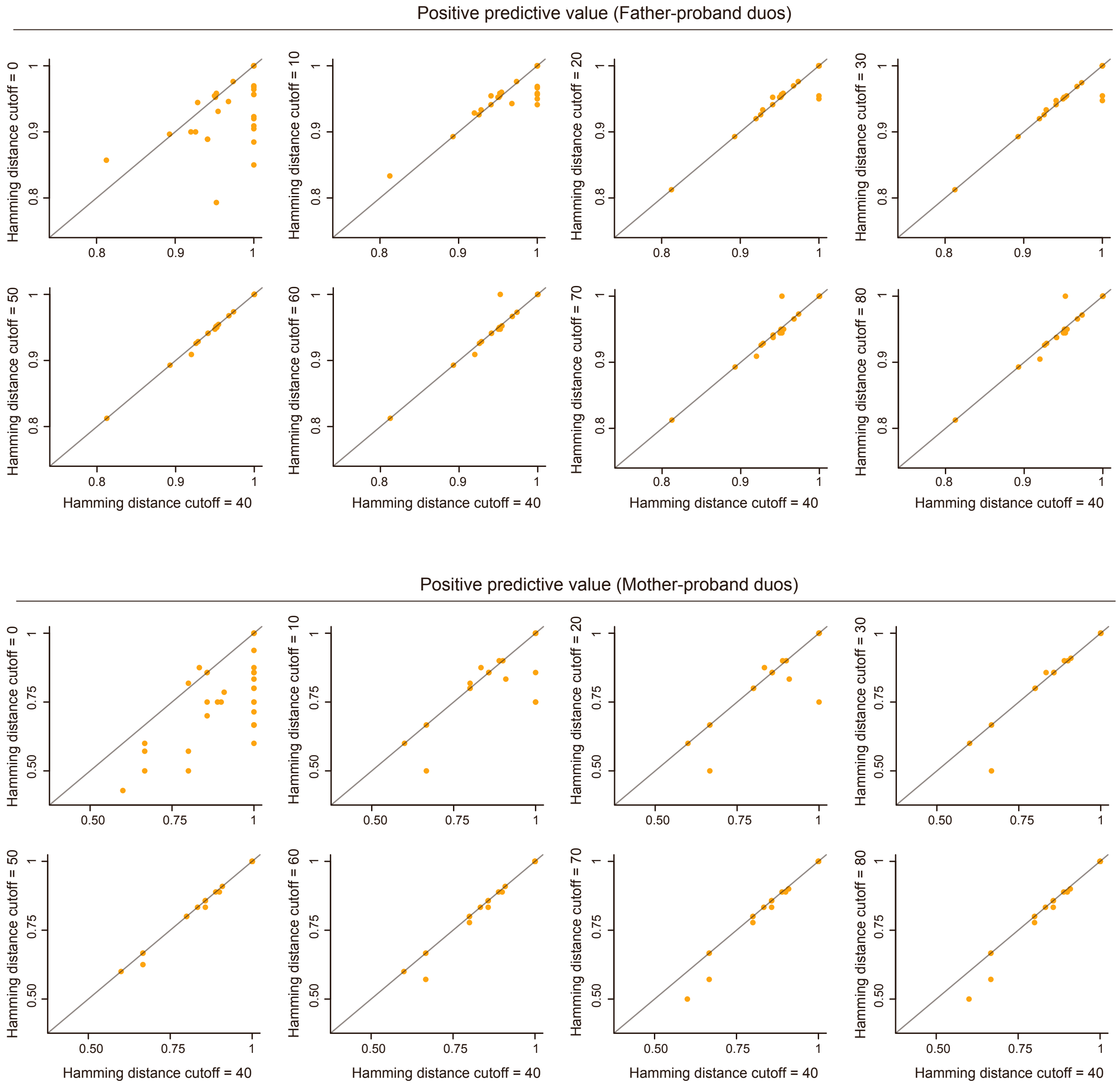

Figure S14. The impact of the Hamming distance threshold for determining dissimilarity between a pair of haplotype blocks on the positive predictive value. Each point corresponds to a duo. The x-axis shows the positive predictive value (PPV) when using a Hamming distance threshold of 40 (default), and the y axis shows the PPV when using different Hamming distance thresholds. There is some degree of overplotting, due to duos that have the same or highly similar PPV.

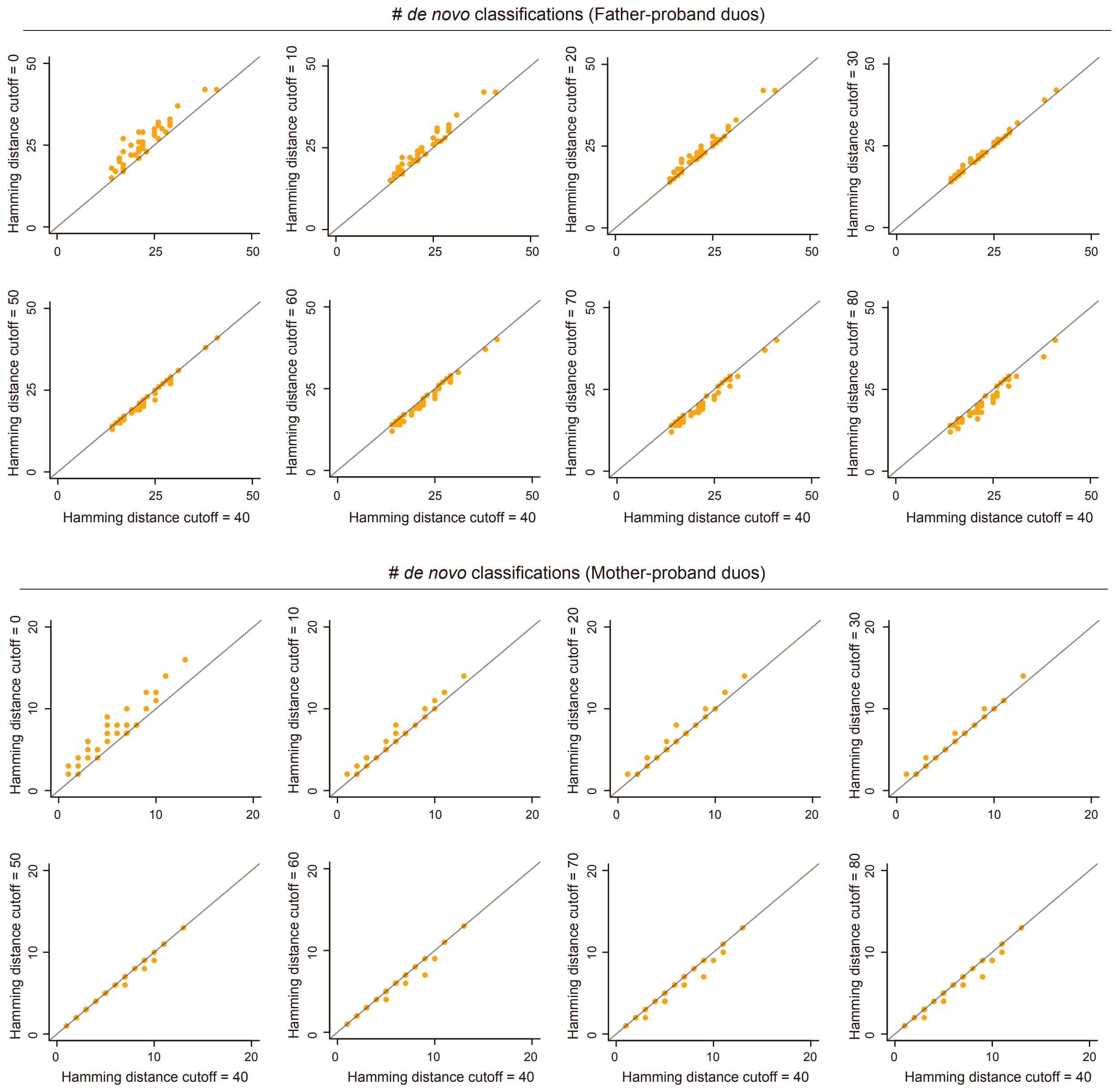

Figure S15. The impact of the Hamming distance threshold for determining dissimilarity between a pair of haplotype blocks on the number of *de novo* classifications. Like Figure S14, but depicting the number of *de novo* classifications instead of the PPV.

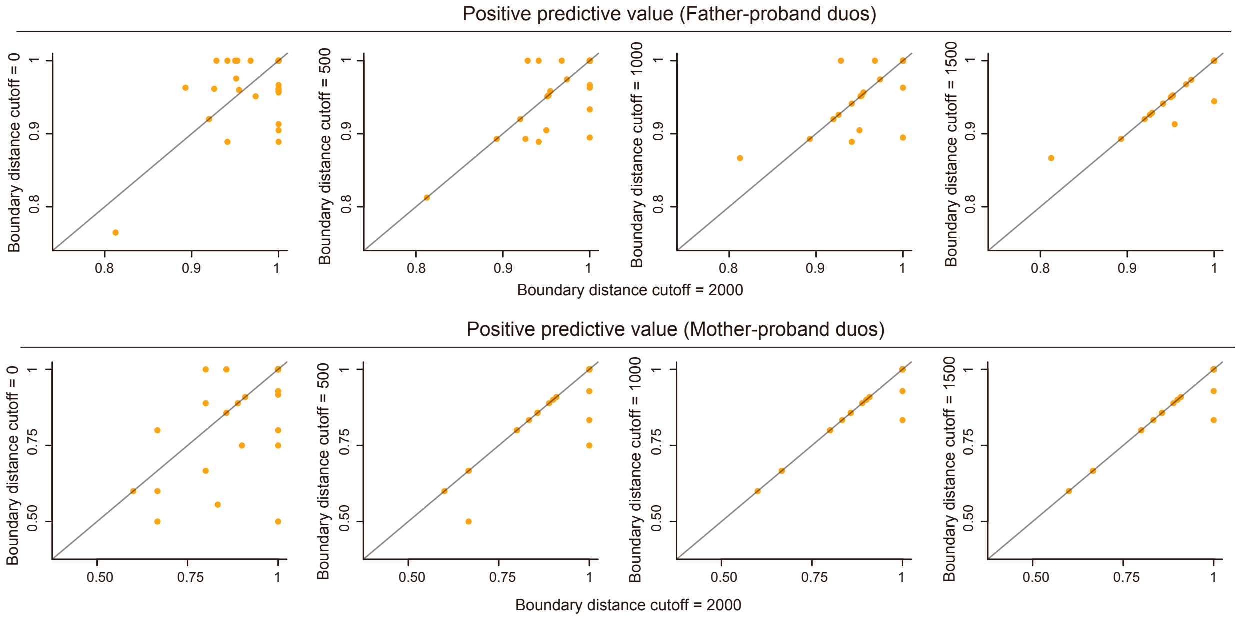

Figure S16. The impact of the distance threshold from haplotype block boundaries on the positive predictive value. Like Figure S14, but showing the impact of the threshold for the distance from the haplotype block boundaries (start/end coordinates) on the PPV.

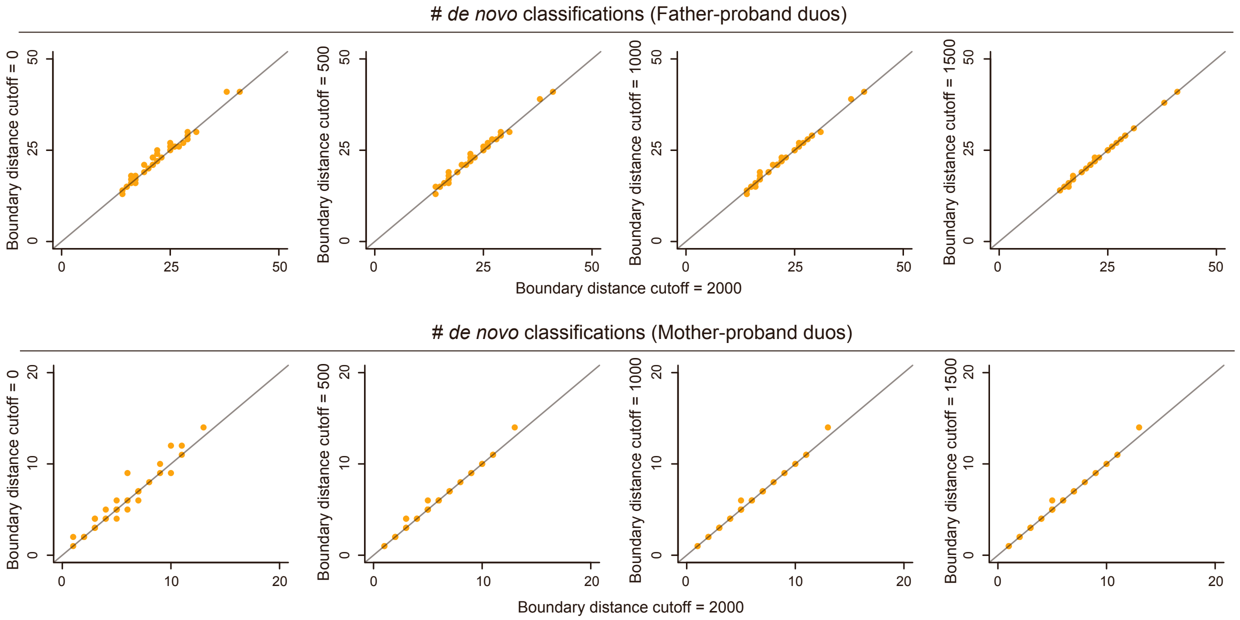

Figure S17. The impact of the distance threshold from haplotype block boundaries on the number of *de novo* classifications. Like Figure S15, but showing the impact of the threshold for the distance from the haplotype block boundaries (start/end coordinates) on the number of *de novo* classifications.

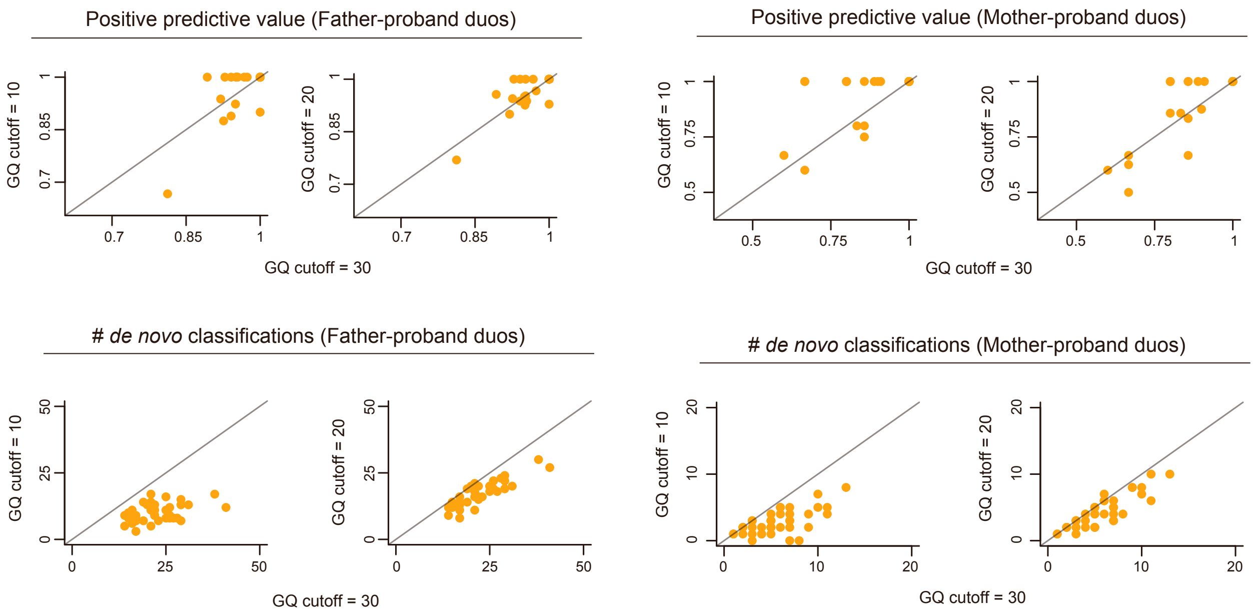

Figure S18. The impact of the phred quality (GQ) threshold at positions surrounding candidate variants on the positive predictive value and the number of *de novo* classifications. Like Figure S14 and Figure S15, but showing the impact of the GQ threshold for positions surrounding the candidate variant (that is, positions which determine the Hamming distance) on the PPV and the number of *de novo* classifications.

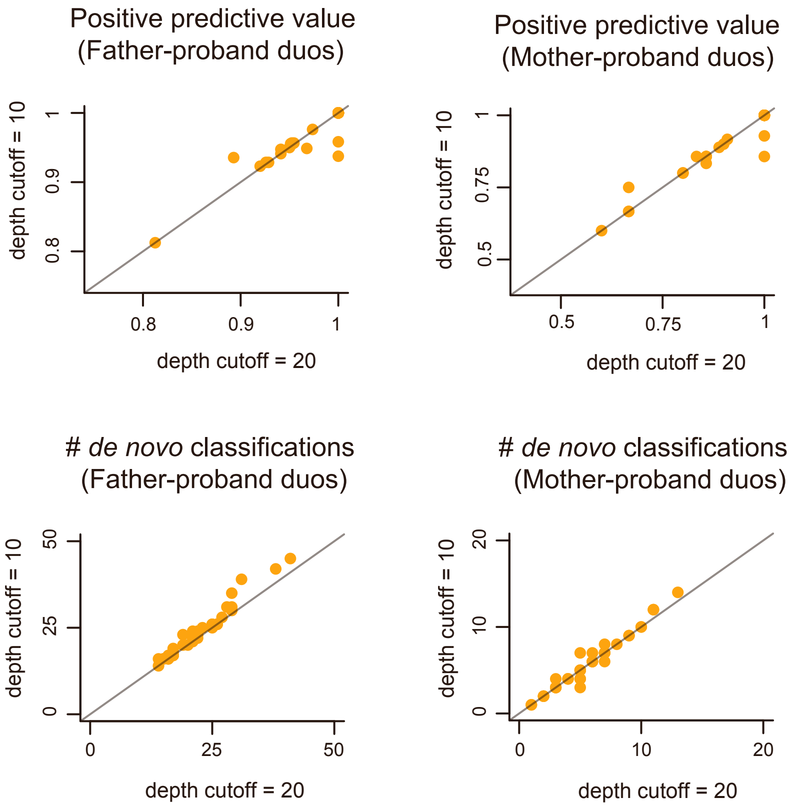

Figure S19. The impact of the sequencing depth threshold at positions surrounding candidate variants on the positive predictive value and the number of *de novo* classifications. Like Figure S18, but showing the impact of the sequencing depth threshold for positions surrounding the candidate variant (that is, positions which determine the Hamming distance) on the PPV and the number of *de novo* classifications.

Supplemental Tables

List of Supplemental Tables

1. **Table S 1 [Available as excel spreadsheet]**: Information (including ancestry, age of parents at childbirth, sequencing depth, and read length) of the participants comprising the 104 trios.

2. **Table S 2**: *duoNovo* Parameter thresholds for genome-scale vs targeted variant analysis (shown below).

3. **Table S 3 [Available as excel spreadsheet]**: List of variants classified as *de novo* by *duoNovo* from the 74 duos of the UCI-GREGoR case set. Includes ENSEMBL annotations based on location with respect to annotated genes, functional consequences for coding variants, variant frequency in different gnomAD populations, CADD Phred score, and spliceAI scores.

Table S 2. Parameter thresholds for genome-scale vs targeted variant analysis.

Parameter	Genome-scale analysis	Targeted variant analysis
Sequencing depth threshold	20	10
GQ threshold	30	20
Hamming distance criterion for hap. block similarity	$= 0$	≤ 2
Hamming distance criterion for hap. block dissimilarity	> 40	> 5
Distance from hap. block boundary threshold (bp)	2000	0
